# A Comprehensive Review of the Phytochemistry and Therapeutic Efficacy of *Viola yedoensis* Makino

**DOI:** 10.3390/molecules30091922

**Published:** 2025-04-25

**Authors:** Shuang Wang, Congcong Shen, Shengyu Zhang, Han Di, Yanhong Wang, Feng Guan

**Affiliations:** 1 School of Pharmacy, Heilongjiang University of Chinese Medicine, 24 Heping Road, Xiangfang District, Harbin 150040, China; swhljucm@163.com (S.W.); scc16637647314@163.com (C.S.); tt13892638691@163.com (S.Z.); 15846488827@163.com (H.D.); 2 Key Laboratory of Basic and Application Research of Beiyao, Ministry of Education, Heilongjiang University of Chinese Medicine, 24 Heping Road, Xiangfang District, Harbin 150040, China

**Keywords:** *Viola yedoensis* Makino, botany, traditional uses, phytochemistry, pharmacology, quality control

## Abstract

*Viola yedoensis* Makino (*V. yedoensis*), a perennial herb in the Violaceae family, is recognized for its violet flowers and has a longstanding role in ethnomedicine for treating various inflammatory diseases, such as boils, furuncles, carbuncles, and both acute and chronic hepatitis, among others. A comprehensive literature review was conducted utilizing resources including the Chinese Pharmacopoeia, Flora of China, Web of Science, PubMed, Baidu Scholar, Google Scholar, and China National Knowledge Infrastructure (CNKI). This paper serves as the inaugural comprehensive review of the latest findings regarding the botany, traditional applications, phytochemistry, pharmacological properties, quality control, and prospective uses of *V. yedoensis*. The objective is to provide a robust foundation for future research and to suggest novel avenues for exploring its potential applications. To date, 162 chemical constituents have been isolated from *V. yedoensis*, with flavonoids and coumarins identified as particularly abundant. These compounds exhibit promising activities, including anti-inflammatory, anti-pyretic, anti-viral, anti-tumor, anti-lung injury, anti-liver injury, anti-bacterial, anti-coagulant, anti-complement, and anti-oxidant properties. Despite considerable advancements in fundamental research on *V. yedoensis*, further investigations are required to elucidate the underlying mechanisms of action and to discover additional uncharacterized compounds. This review underscores the plant’s significant development potential, highlighting the necessity for more in-depth exploration.

## 1. Introduction

*Viola yedoensis* Makino (*V. yedoensis*) is a small perennial herb characterized by violet flowers that is predominantly found in subtropical and temperate regions of the Northern Hemisphere, including China, Japan, and Korea [1]. As a significant member of the *Viola* genus within the Violaceae family, it ranks among the most widely utilized natural medicinal plants, with a longstanding history in empirical medicine [2]. In China, the dried whole plant (including the roots), referred to as “Zi Hua Di Ding”, is a well-established component of traditional Chinese medicine (TCM) [3,4]. It is traditionally employed as an anti-pyretic and detoxifying agent, effectively addressing various inflammatory conditions such as boils, furuncles, carbuncles, and both acute and chronic hepatitis [5,6]. The therapeutic uses of *V. yedoensis* have been documented in historical texts, with multiple entries in the Chinese Pharmacopoeia, the first of which appeared in the 1963 edition. The 2020 edition of the Pharmacopoeia of the People’s Republic of China (ChP, 2020) [7] specifies that the dried whole plant is effective in clearing heat, detoxifying, cooling blood, and reducing swelling, with external applications also indicated for treating bruises and snake bites [8].

As one of the most medicinally valuable species within the genus *Viola*, *V. yedoensis* is distinguished from other closely related species by its unique chemical constituents and diverse pharmacological activities. To date, about 162 compounds have been identified in *V. yedoensis*, representing a broad spectrum of chemical groups, including flavonoids, coumarins, terpenoids, phenolic acids, alkaloids, and cyclopeptides [9,10]. Compared to other species in the *Viola* genus, such as *Viola odorata* (*V*. *odorata*), *Viola tianshanica* (*V*. *tianshanica*), and *Viola mandshurica* (*V*. *mandshurica*), *V. yedoensis* contains a higher abundance of key bioactive compounds, particularly flavonoids and coumarins [11]. This chemical richness provides a solid foundation for its wide range of pharmacological activities. Contemporary pharmacological research has demonstrated that extracts of *V. yedoensis* and its chemical constituents exhibit significant pharmacological activities, such as anti-inflammatory, anti-pyretic, anti-viral, anti-tumor, anti-lung injury, anti-liver injury, anti-bacterial, anti-coagulant, anti-complement, and anti-oxidant effects [12]. The volatile oil and total flavonoids of *V. yedoensis* exhibit significantly stronger anti-bacterial effects against various pathogenic bacteria compared to similar extracts from *V*. *odorata*. Moreover, its characteristic cyclopeptide components further enhance anti-bacterial activity by disrupting the membrane structure of pathogens. In addition, compared to *V*. *tianshanica*, whose pharmacological effects are primarily limited to anti-oxidant and anti-inflammatory activities, *V. yedoensis* demonstrates a multi-target mechanism of action, offering broader therapeutic potential for the treatment of complex diseases. The remarkable diversity of its chemical constituents and pharmacological activities gives *V. yedoensis* a distinct advantage over other species within the *Viola* genus, highlighting its greater medicinal value in clinical applications [2,11].

Clinically, *V. yedoensis* is used in TCM for treating various inflammatory diseases, including bronchitis, hepatitis, acute nephritis, appendicitis, and enteritis, demonstrating notable therapeutic efficacy [13]. Recent studies particularly highlight its potent anti-inflammatory properties, showing protective effects on the skin by significantly reducing inflammatory cytokine levels and alleviating symptoms of atopic dermatitis (AD). Whether utilized independently or in compound formulations, *V. yedoensis* is an essential herb in the management of AD and broader TCM applications [14].

The medicinal value of *V. yedoensis* is substantial, offering a wealth of natural resources. The bioactive compounds derived from this plant exhibit high bioavailability and therapeutic potential, making it a promising source for drug discovery and the development of new treatments. Despite numerous studies addressing specific aspects of its phytochemistry and pharmacology, a comprehensive and current review has been lacking. This paper aims to bridge that gap by presenting the first extensive review of the botany, traditional uses, phytochemistry, pharmacological activities, quality control, and potential applications of *V. yedoensis*, thereby establishing a robust scientific foundation for future research on this valuable plant.

## 2. Botany

### 2.1. Plant Sources

**Figure 1:**
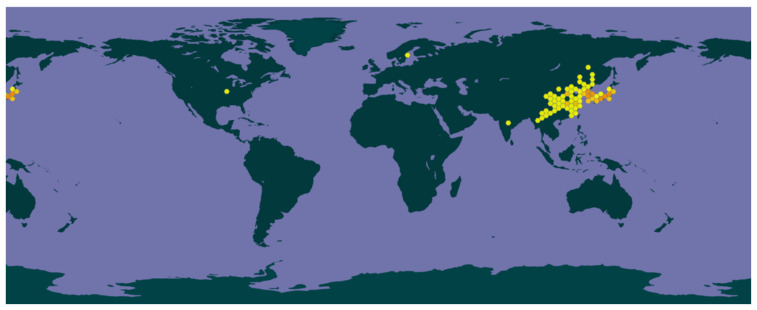
General geographical distribution of *V. yedoensis* in the world (www.gbif.org).

*V. yedoensis*, a species within the genus *Viola* of the *Violaceae* family, is extensively distributed across various regions of China, including Heilongjiang, Jilin, Liaoning, Inner Mongolia, Hebei, Shanxi, Shaanxi, Gansu, Shandong, Jiangsu, Anhui, Zhejiang, Jiangxi, Fujian, Taiwan, Henan, Hubei, Hunan, Guangxi, Sichuan, Guizhou, and Yunnan. Its distribution extends beyond China to Korea, Japan, and the Russian Far East. This species thrives in environments characterized by ample sunlight and exhibits strong adaptability to moist conditions. *V. yedoensis* is commonly found in diverse ecological settings, such as fields, wastelands, grassy slopes, forest edges, and shrublands. The geographical distribution of *V. yedoensis* worldwide was obtained from the GBIF online database (www.gbif.org, illustrated in Figure 1).

### 2.2. Plant Traits

**Figure 2:**
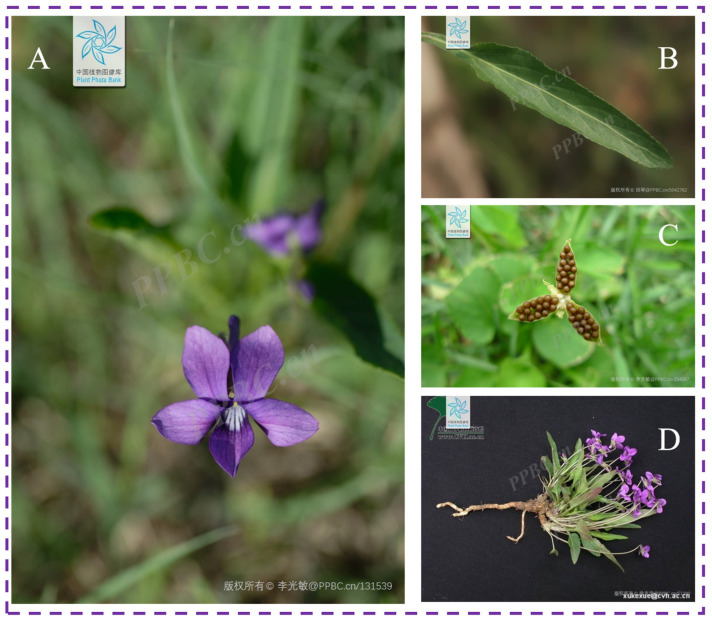
Plant morphology of *V. yedoensis*. (**A**) Flowers, (**B**) leaves, (**C**) flowers, (**D**) dry medicinal parts. (The plant images in this article are from the open resources of Plant Photo Bank of China, PPBC).

As a perennial herb, *V. yedoensis* exhibits notable morphological characteristics. Photographs of the plant are provided in Figure 2. According to records from the Flora of China (http://www.iplant.cn), *V. yedoensis* lacks an aerial stem, with a height ranging from 4 to 14 cm during the non-fruiting period and exceeding 20 cm during fruiting. The rhizome is short, vertically oriented, and light brown, measuring between 4 and 13 mm in length and 2 to 7 mm in diameter, with closely spaced nodes. Surrounding the base of the rhizome are small, slender roots that range in color from light brown to nearly white. The plant produces numerous basal leaves arranged in a rosette pattern. The lower leaves are smaller, triangular-ovate, or narrowly ovate, while the upper leaves are larger, exhibiting oblong, narrowly ovate-lanceolate, or oblong-ovate shapes, measuring 1.5 to 4 cm in length and 0.5 to 1 cm in width. The leaf apex is typically rounded or blunt, with bases that are truncate, cuneate, or occasionally subcordate. The leaf margins are slightly serrated with rounded teeth. Both leaf surfaces may be glabrous or sparsely covered with short, fine hairs, particularly noticeable along the veins on the underside. During the fruiting stage, leaves expand to lengths exceeding 10 cm and widths reaching up to 4 cm. The petioles of *V. yedoensis* are typically one to two times longer than the leaf blades during the flowering period and may feature extremely narrow, wing-like structures along the upper edge. During the fruiting stage, the petioles further elongate, often exceeding 10 cm, with the winged structures becoming more pronounced. Petioles may be glabrous or covered with fine, short hairs. The stipules are membranous, pale white or light green, measuring 1.5 to 2.5 cm in length. Approximately two-thirds to four-fifths of the stipules are fused with the petiole, while the free portion is linear-lanceolate. The margins of the stipules may be sparsely fringed with glandular, fimbriate teeth or may appear nearly entire. The flowers of *V. yedoensis* are of moderate size, typically violet or pale purple, although some may be white with lighter throats streaked with purple. The plant produces numerous slender peduncles that are equal to or slightly longer than the leaves and may be either glabrous or covered with short, soft hairs. Each peduncle typically bears two linear bracts near the middle. The sepals are lanceolate or ovate-lanceolate, measuring 5 to 7 mm in length, with gradually tapering apices. The basal appendages are short, measuring 1 to 1.5 mm, with rounded or truncate ends, and their margins may be membranous and white. The sepals can be either glabrous or covered with short, soft hairs. The petals are obovate or oblong-obovate, with lateral petals measuring 1 to 1.2 cm in length, which may be glabrous or bearded on the inner surface. The lower petal, including the spur, is longer, reaching 1.3 to 2 cm, and is marked with purple veins on the inner surface. The spur is slender and tubular, measuring 4 to 8 mm in length, with a rounded tip. The anthers are approximately 2 mm long, featuring appendages at the top of the connective portion that measure about 1.5 mm. The spurs of the two lower stamens are also slender and tubular, measuring 4 to 6 mm, with slightly narrower ends. The ovary is ovate and glabrous, while the style is club-shaped, slightly longer than the ovary, with a slightly curved base. The stigma is triangular, with slightly thickened edges along the sides and rear, forming subtle ridges. The top of the stigma is somewhat flattened, featuring a short beak at the front. The capsule is oblong, measuring 5 to 12 mm in length, and is glabrous. The seeds are ovoid, about 1.8 mm in length, and pale yellow in color. *V. yedoensis* typically flowers and fruits from mid-April through September.

## 3. Traditional Uses

*V. yedoensis*, commonly referred to in China as “Zi Hua Di Ding”, has a rich medicinal history in TCM spanning several centuries [3]. Its therapeutic properties were first documented in the Ming dynasty by Li Shizhen in the Compendium of Materia Medica, which emphasizes its abilities to clear heat, detoxify, cool the blood, and reduce swelling. Additionally, Qian Jin Yao Fang notes that *V. yedoensis* can “go through the menstrual flow to vent fire, dissipate swelling, and disinfect”, while also describing its functions to “invigorate the liver, dry the spleen, calm heat of blood, and remove stagnant dampness”, indicating its liver-protective effects [15]. Historical records in folk culture further illustrate its use as a traditional remedy for various ailments. *V. yedoensis* has been widely employed to treat conditions such as swelling, ulcers, furuncles, and carbuncles [16]. According to ChP, 2020, the recommended dosage for *V. yedoensis* is 15–30 g, typically prepared as an infusion for treating carbuncles, abscesses, erysipelas, and snake bites. Notably, its traditional applications are closely linked to inflammatory conditions. In TCM theory, each herbal medicine possesses distinct flavors and properties. *V. yedoensis* is characterized as bitter and spicy, with a cold nature. It exerts stimulating effects on specific meridians, particularly the heart and liver meridians. Due to its positive influence on these pathways, *V. yedoensis* is utilized for clearing heat, removing toxicity, cooling the blood, and reducing swelling [15,17]. In contemporary traditional medicine practice, the theories of flavor and meridian pathways significantly inform the clinical application of herbal remedies [18,19,20].

Clinically, *V. yedoensis* has demonstrated significant efficacy in treating various inflammatory diseases, including bronchitis, acute and chronic hepatitis, acute nephritis, appendicitis, and enteritis [21]. Additionally, it has shown remarkable effectiveness in addressing purulent and infectious diseases. Notably, accumulating clinical evidence reveals that *V. yedoensis* excels in treating skin conditions, particularly AD and allergic skin reactions. The *V. yedoensis* formula (VYAC), a traditional herbal compound consisting of *V. yedoensis*, *Sophora flavescens* Ait., and *Dictamnus dasycarpus* Turcz., is currently widely utilized in clinical settings to alleviate symptoms and associated inflammatory responses of AD induced by 2,4-dinitrochlorobenzene (DNCB) [14]. In summary, modern research supports many traditional uses of *V. yedoensis*, particularly its anti-inflammatory and antiviral properties. However, further investigation is warranted regarding its other traditional applications, such as its roles as an expectorant and diuretic. Therefore, exploring the relationship between *V. yedoensis*’s traditional uses and its reported pharmacological activities merits additional study.

## 4. Phytochemistry

*V. yedoensis* derives its medicinal properties from a diverse array of phytochemical constituents. To date, numerous compounds have been isolated and identified from the whole herbs of this species, including flavonoids (**1**–**39**), coumarins (**40**–**65**), terpenoids (**66**–**82**), phenolic acids (**83**–**91**), alkaloids (**92**–**109**), cyclotides (**122**–**162**), and other constituents (**110**–**121**). Among these, flavonoids and coumarins stand out as the most representative components, exhibiting multiple bioactivities and serving as a critical material basis for their anti-pyretic and detoxifying effects. Detailed phytochemical information is summarized in Table 1.

### 4.1. Flavonoids

**Figure 3:**
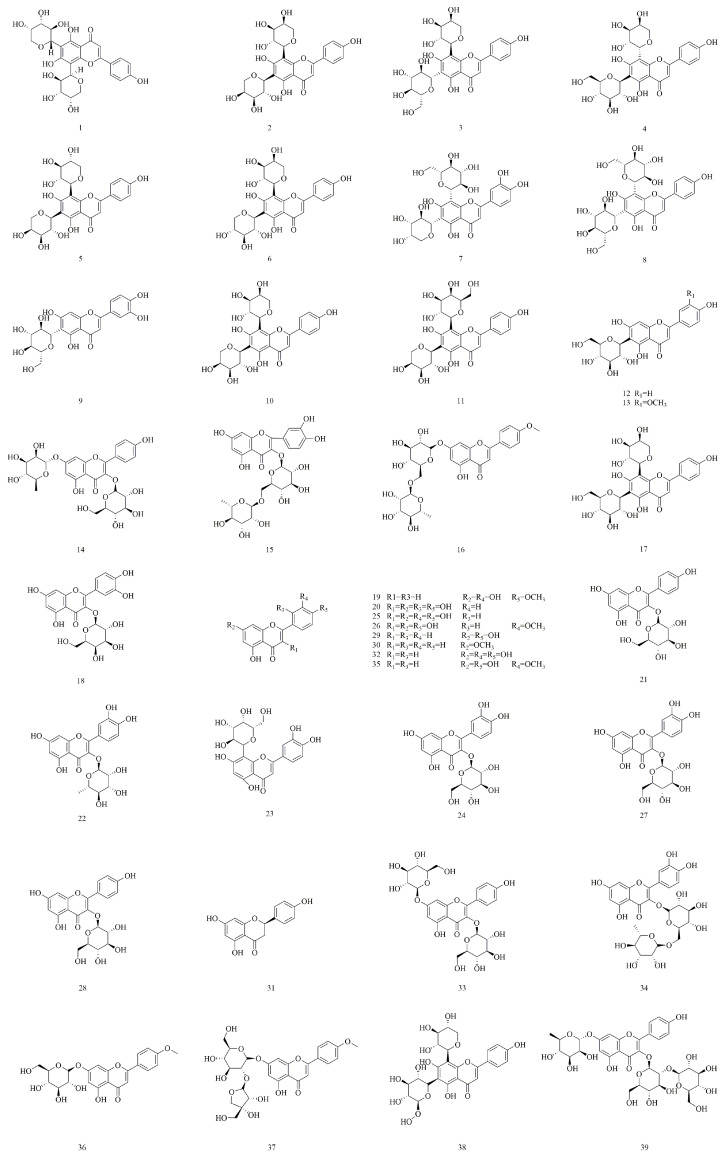
Structures of flavonoids in *V. yedoensis*.

Flavonoids, a class of natural polyphenols, play a significant role in human health [33,34,35]. Previous studies have identified flavonoids as the primary bioactive constituents of *V. yedoensis* due to their high concentrations in the plant. A total of 39 flavonoid compounds have been isolated and categorized into four subclasses: flavones, flavonols, dihydroflavones, and flavonoid glycosides. The flavone subclass includes compounds such as diosmetin (**19**), apigenin (**29**), techtochrysin (**30**), luteolin (**32**), and chrysoeriol (**35**). The concentrations of these compounds in *V. yedoensis* are significantly higher than those found in other species of the *Viola* genus, such as *V. odorata* and *V. tianshanica*. Notably, luteolin (**32**) is a promising natural flavonoid found in *V. yedoensis*, with potential applications in treating various human diseases, indicating significant clinical prospects [36]. The flavonol subclass comprises morin (**20**), quercetin (**25**), and isorhamnetin (**26**), with quercitrin (**22**) recognized as a relatively common dietary flavonoid [37]. The dihydroflavone identified is naringenin (**31**). Most flavonoids in *V. yedoensis* are present in glycosylated forms. For example, quercetin-3-*O*-β-d-glucoside (**27**) and kaempferol-3-*O*-β-d-glucoside (**28**) typically bind to sugar moieties such as glucose or arabinose to form glycosides. This glycosylation enhances the stability and bioavailability of these compounds within the plant. Moreover, the glycosylation sites and linkage pattern of apigenin-6,8-di-*C*-α-l-arabinopyranoside (**10**) are rare among other species within the same genus. This metabolic profile is more pronounced in *V. yedoensis*, whereas flavonoids in other species of the same genus may predominantly exist in non-glycosylated forms. In particular, the flavonoid biosynthesis and metabolic pathways in *V. yedoensis* also differ from those in related species. *V. yedoensis* may possess a specific enzymatic system that plays a key role in its metabolic processes, potentially facilitating the synthesis of unique flavonoid compounds and promoting their accumulation in glycosylated forms. The structures of these flavonoids (**1**–**39**) are presented in Figure 3.

### 4.2. Coumarins

**Figure 4:**
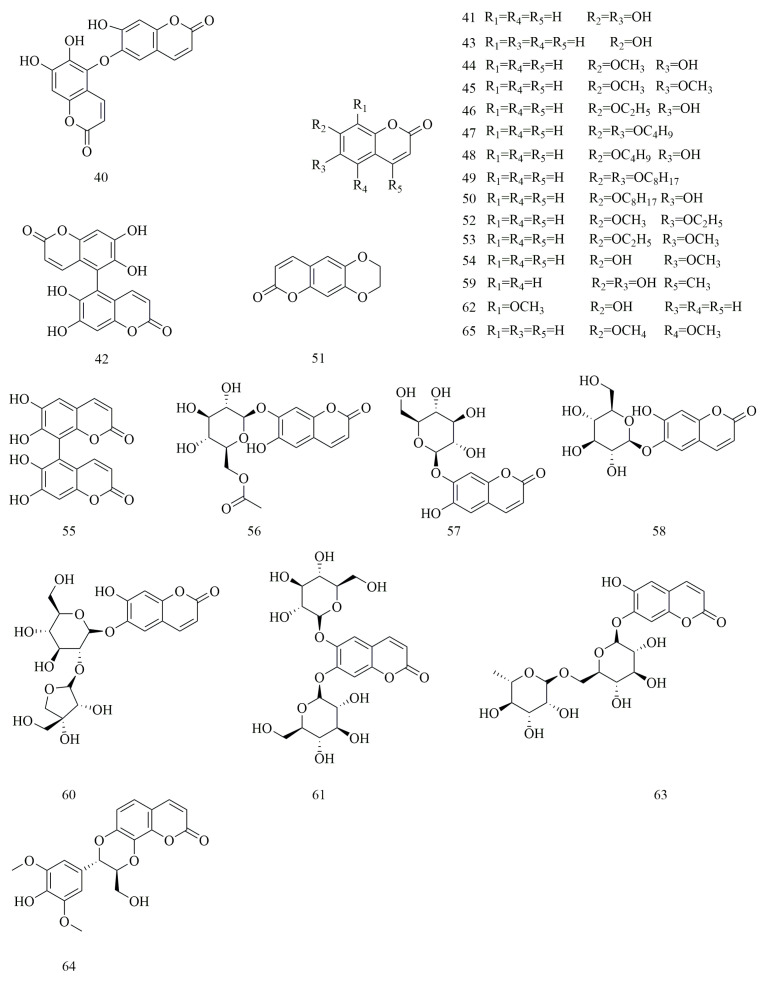
Structures of coumarins in *V. yedoensis*.

Coumarins, a class of naturally occurring heterocyclic compounds, are frequently utilized in the synthesis of various biologically and pharmacologically active substances [38,39,40]. In *V. yedoensis*, coumarin compounds represent a significant class of constituents, with 26 compounds (**40**–**65**) isolated and identified. Notable examples include dimeresculetin (**40**), 6,6’,7,7’-tetrahydroxy-5,8’-biscoumarin (**55**), and esculin (**58**), which have been characterized using mass spectrometry (MS) and nuclear magnetic resonance (NMR). The first two compounds exhibit significant anticoagulant properties, suggesting potential clinical applications in treating cardiovascular and cerebrovascular diseases. Conversely, the latter two demonstrate notable inhibitory activity against HCV protease. Dimeresculetin (**40**) is also a coumarin compound uniquely found in *V. yedoensis*. Additionally, four glycoside forms of coumarin have been isolated: euphorbetin (**42**), prionanthoside (**56**), cichoriin (**57**), and 6-hydroxy-coumarin-7-*O*-α-l-rhamnosyl-(1→6)-*O*-β-d-glucoside (**63**). The remaining coumarin compounds are categorized as simple coumarins. Esculetin (**41**), a naturally occurring dihydroxycoumarin in *V. yedoensis*, is present at the highest concentration among species of the same genus and is recognized as a key component for quality control standards in the Chinese Pharmacopoeia for this plant (Figure 4).

### 4.3. Terpenoids

**Figure 5:**
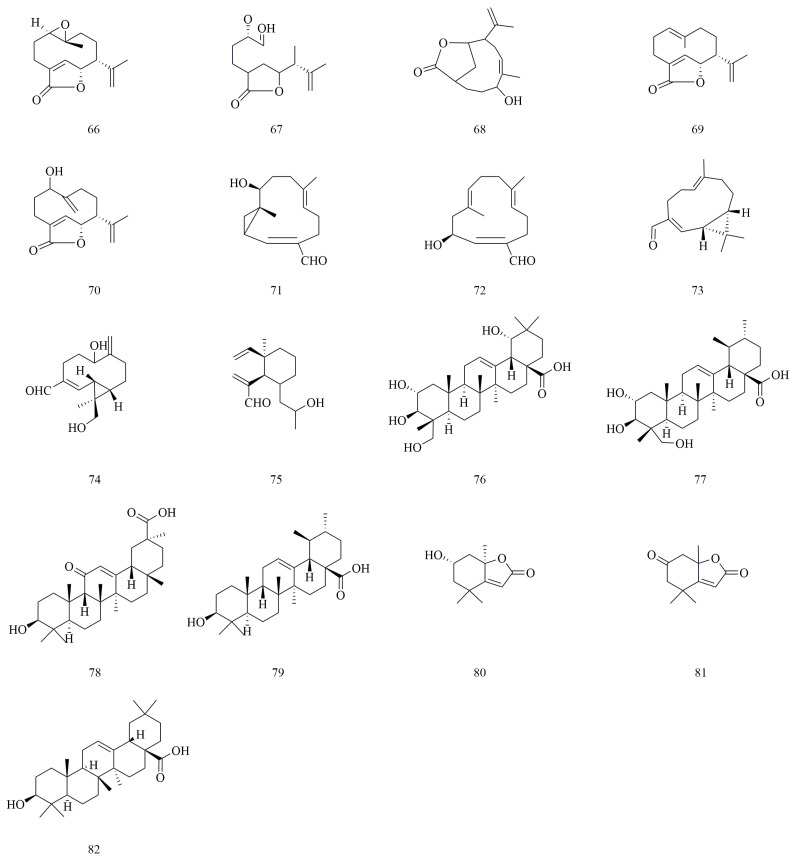
Structures of terpenoids in *V. yedoensis*.

Terpenoids, which comprise one of the largest and most chemically diverse classes of natural secondary metabolites, are formed from isoprene units and exhibit a wide range of biological properties [41,42]. From *V. yedoensis*, 16 terpenoids have been isolated, with compounds **66**–**75** classified as sesquiterpenes. Yedoensins A (**66**) and yedoensins B (**67**) are sesquiterpenoids that were first isolated from *V. yedoensis* in 2015 and are unique to this species, having not been found in other species of the same genus. Loliolide (**80**) and dehydrololiolide (**81**) belong to the monoterpene lactone class. Additionally, *V. yedoensis* contains five triterpenoid compounds, including oleanane-type triterpenes such as arjungenin (**76**), 18-β-glycyrrhetinic acid (**78**), and oleanolic acid (**82**), alongside common ursane-type triterpenes like asiatic acid (**77**) and ursolic acid (**79**). The chemical structures of terpenoids **66**–**82** are illustrated in Figure 5.

### 4.4. Phenolic Acids

**Figure 6:**
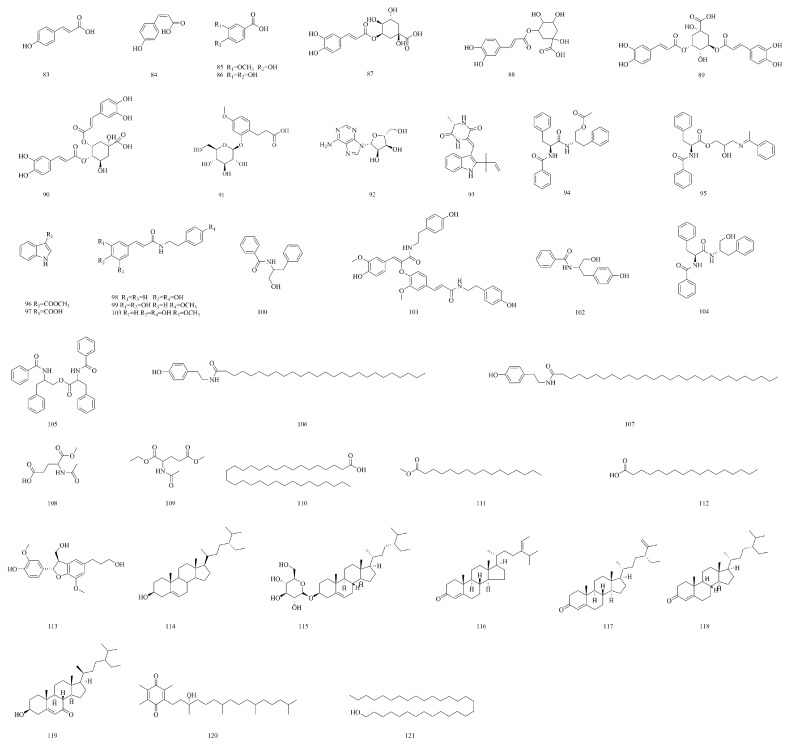
Structures of phenolic acids, alkaloids, and other compounds in *V. yedoensis*.

Despite the widespread occurrence of phenolic acids in various plants [43], only nine phenolic acids (**83**–**91**) have been documented in *V. yedoensis* to date (Figure 6). Among these, the representative phenolic acids include *cis*-*p*-coumaric acid (**84**), vanillic acid (**85**), protocatechuic acid (**86**), and chlorogenic acid (**88**). Notably, protocatechuic acid (**86**) is present in *V. yedoensis* at the highest concentration observed among all *Viola* species. All these compounds have been successfully isolated from the plant’s ethanol extract.

### 4.5. Alkaloids

Alkaloids, a class of naturally occurring nitrogen-containing organic compounds, possess a broad spectrum of health-related applications [44,45]. A total of 20 alkaloids have been reported in *V. yedoensis*, primarily identified from its 95% ethanol extract using various column chromatography techniques, including silica gel (Qingdao Ocean Chemical Factory, Qingdao, China) and Sephadex LH-20 (Pharmacia, Stockholm, Sweden). Notably, *N*-(4-hydroxyphenethyl) hexacosanamide (**106**) and *N*-(4-hydroxyphenethyl) octacosanamide (**107**) have been identified exclusively in *V. yedoensis* and have not been detected in other species, such as *V. odorata* or *V. tianshanica*. These alkaloids have been shown to exhibit significant anti-complement activity, with CH_50_ and AP_50_ values ranging from 0.12 to 0.33 g/L and 0.22 to 0.50 g/L, respectively (Figure 6). The anti-complement activity of cannabisin F (**101**) in *V. yedoensis* (CH_50_ = 0.16 g/L) is significantly higher than that of the indole alkaloids found in *V. odorata* [11].

### 4.6. Cyclotides

The genus *Viola* is a prominent family of cyclotide-producing plants [46,47], with cyclotides being cysteine-rich peptides typically comprising 28 to 37 amino acids [48]. As a significant member of this genus, *V. yedoensis* serves as an essential source of cyclotides, with over 41 identified to date. Cycloviolacin Y1–Y5 (**140**–**144**) and varv E (**147**) are unique constituents of *V. yedoensis*, characterized by distinctive amino acid sequences that have not been reported in other *Viola* species. Recent research has increasingly highlighted the antiviral properties of these cyclotides, further emphasizing their therapeutic potential [2,11]. Compared with other *Viola* species, the cyclotides in *V. yedoensis* exhibit significantly greater structural complexity and functional diversity. Future research should focus on elucidating the key enzymatic mechanisms involved in their biosynthetic pathways to advance applications in synthetic biology. Table 2 presents the names and amino acid sequences of these cyclotides, distinguishing them from other small molecular compounds.

### 4.7. Other Compounds

Beyond the prominent natural product families previously mentioned, *V. yedoensis* is abundant in a diverse array of additional components. The fatty acids present include lacceroic acid (**110**), methyl palmitate (**111**), and stearic acid (**112**). The lignan rel-(2α,3β)-7-*O*-methylcedrusin (**113**) is also present, alongside sterols such as β-sitosterol (**114**), daucosterol (**115**), stigmasta-4,24(28)-dien-3-one (**116**), stigmasta-4,25-dien-3-one (**117**), β-sitostenone (**118**), and (24*R*)-3β-hydroxy-ethylcholest-5-en-7-one (**119**). Furthermore, α-tocopherol-quinone (**120**), an oxidation product of vitamin E (α-tocopherol), and triacontanol (**121**), a long-chain fatty alcohol, have been identified. The structures of these compounds (**110**–**121**) are illustrated in Figure 6.

## 5. Pharmacology

**Figure 7:**
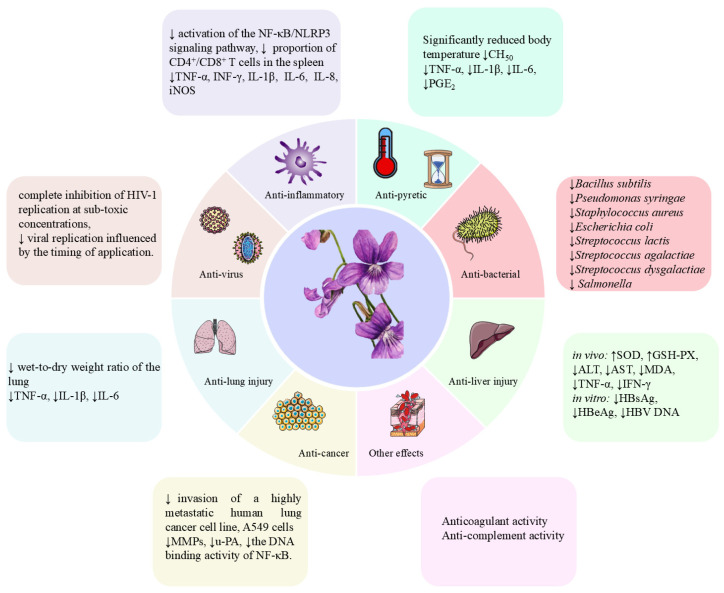
Pharmacological activities of *V. yedoensis*. (↑): improve or promote. (↓): inhibit or reduce.

*V. yedoensis*, a traditional Chinese herbal medicine prevalent throughout China, has undergone extensive investigation in modern pharmacological research, elucidating many of its traditional benefits. This plant exhibits a broad spectrum of pharmacological activities, including anti-inflammatory, anti-pyretic, anti-viral, anti-tumor, anti-lung injury, anti-liver injury, anti-bacterial, anti-coagulant, anti-complement, and anti-oxidant effects. Notably, its traditional applications have laid a foundational basis and provided direction for contemporary pharmacological studies. The diversity of its pharmacological effects further highlights the potential value of these traditional uses. A summary of the various pharmacological activities of *V. yedoensis* is presented in Table 3 and illustrated in Figure 7.

### 5.1. Anti-Inflammatory Effects

Inflammatory disorders, which arise from various factors, rank among the most common conditions encountered in daily life, with severe inflammation potentially leading to fatal outcomes [57,58]. Clinical manifestations of inflammation primarily include swelling and localized heat pain [59]. Traditionally, *V. yedoensis* has been utilized in ethnomedicine for its heat-clearing, detoxifying properties and ability to cool the blood and reduce swelling, which suggest significant anti-inflammatory activity. A recent study evaluated the anti-inflammatory effects of *V. yedoensis* on the spleen and thymus of broiler chickens exposed to heat stress. Findings indicated that under heat stress conditions, serum levels of immunoglobulins IgA, IgG, and IgM, along with the levels of Newcastle disease (ND) and infectious bursal disease (IBD) antibodies, were significantly reduced. Concurrently, the expression levels of inflammatory factors interleukin-1β (IL-1β) and INF-γ in the spleen and thymus increased, signifying a pronounced inflammatory response. Notably, the addition of *V. yedoensis* to the feed, particularly at doses of 1.5% and 4.5%, significantly inhibited the expression of these inflammatory factors. Treatment groups receiving *V. yedoensis* exhibited reduced levels of IL-1β and INF-γ compared to the heat stress group, alongside marked decreases in histopathological damage to the spleen and thymus, including reduced inflammatory cell infiltration and tissue structural damage [51]. These results suggest that *V. yedoensis* effectively mitigates inflammatory damage to the immune organs of broiler chickens induced by heat stress within a specific dosage range, especially at 1.5% and 4.5%. This discovery supports the potential application of *V. yedoensis* as an anti-inflammatory feed additive. In a separate study, Zhao et al. investigated the modulatory effects of *V. yedoensis* on lipopolysaccharide (LPS)-induced intestinal inflammation in broiler chickens. An intestinal injury model was established via intraperitoneal injection of LPS, and the potential alleviating effects of *V. yedoensis* were assessed by incorporating varying doses (0.5%, 1.5%, and 4.5%) into the feed. The results demonstrated that *V. yedoensis* significantly reduced levels of inflammatory factors in the intestines of broiler chickens following LPS stimulation, including tumor necrosis factor-α (TNF-α), IL-1β, IL-8, NLRP3, Caspase-1, MyD88, and TLR4 [52]. Additionally, it effectively inhibited the activation of the NF-κB/NLRP3 signaling pathway. These results indicate that *V. yedoensis* alleviates LPS-induced intestinal inflammation in broiler chickens by modulating critical inflammatory signaling pathways.

Recent research has thoroughly elucidated the anti-inflammatory properties of the *V. yedoensis* anti-itch complex (VYAC) in RBL-2H3 mast cells. Experimental findings indicated that the anti-itch complex exhibited good cell tolerance within a dosage range of 25 to 400 μg/mL, while a higher dosage of 800 μg/mL resulted in significant cytotoxicity. A23187 and PMA served as positive controls to mimic allergic reactions in mast cells. Comprehensive data demonstrated that the VYAC markedly inhibited the mRNA expression of inflammatory cytokines, including TNF-α, IL-1β, interleukin-6 (IL-6), and iNOS, at doses of 100, 200, and 400 μg/mL, with the inhibitory effect becoming more pronounced at higher dosages. Notably, at dosages of 200 μg/mL and 400 μg/mL, the suppression of inflammatory cytokine expression was particularly significant, while no statistically meaningful differences were found at the 100 μg/mL dosage [28]. These observations highlight the potential of natural compounds derived from *V. yedoensis* as candidates for the treatment of inflammation, owing to their substantial efficacy and safety profiles.

Importantly, exploring natural products with anti-inflammatory properties is essential for the treatment of AD, a common inflammatory skin condition that severely impacts patients’ quality of life and imposes a significant economic burden on families [60,61]. Fan et al. demonstrated that the ethanol extract of *V. yedoensis* exhibited notable therapeutic effects in a DNCB-induced AD-like skin lesion model in ICR mice. In their study, the extract was administered at doses of 5 mg/day and 10 mg/day, with dexamethasone (DEX) serving as a positive control to evaluate its impact on the DNCB-induced inflammatory response. The results indicated that the ethanol extract of *V. yedoensis* dose-dependently alleviated AD-like lesions, resulting in reduced ear swelling, the suppression of epidermal thickening, and decreased inflammatory cell infiltration. Furthermore, the extract lowered serum levels of IL-1β, IL-6, and TNF-α, as well as the proportion of CD4^+^/CD8^+^ T cells in the spleen, suggesting therapeutic effects through the inhibition of T cell-mediated immune responses. In lesional skin tissue, the ethanol extract also reduced the expression levels of iNOS and COX-2 in a dose-dependent manner, further confirming its anti-inflammatory properties. Compared to DEX, the ethanol extract of *V. yedoensis* exhibited significant anti-inflammatory activity and may serve as a natural alternative therapy for treating AD. Notably, VYAC has shown promising results in the treatment of AD [22]. Extensive research indicates that these effects are attributable to its ability to reduce levels of pro-inflammatory cytokines, including IL-1β, TNF-α, and IL-18, while enhancing levels of the anti-inflammatory cytokine IL-10. Moreover, VYAC facilitates the polarization of M2 macrophages, further substantiating its anti-inflammatory properties. Notably, the efficacy of high-dose VYAC (600 mg/kg) is comparable to that of the standard treatment drug dexamethasone (2.5 mg/kg). These results suggest that VYAC exerts significant anti-inflammatory effects by activating the JAK2/STAT3 signaling pathway and promoting the polarization of M2 macrophages. Taken together, the traditional use of *V. yedoensis* in treating various inflammatory diseases aligns with its established anti-inflammatory properties. Modern pharmacological research further substantiates its potential clinical value in addressing inflammatory conditions, providing renewed motivation for exploring *V. yedoensis* as a candidate drug for clinical therapy.

### 5.2. Anti-Pyretic Effects

*V. yedoensis* is esteemed for its traditional anti-pyretic properties [62]. Recent investigations have underscored its promising anti-pyretic activity, particularly in an LPS-induced fever model utilizing rabbits. Pan et al. assessed the effects of various doses of *V. yedoensis* extracts, including aqueous extracts (10, 30, 50 mg/kg) and ethanol extracts (10, 30, 50 mg/kg), alongside fractions derived from the ethanol extract, such as petroleum ether (PE, 10, 30, 50 mg/kg), ethyl acetate (EA, 10, 30, 50 mg/kg), and n-butanol (BU, 10, 30, 50 mg/kg). Aspirin (20 mg/kg) served as the positive control. Notably, the diverse fractions of *V. yedoensis* exhibited significant anti-pyretic effects. In the LPS-induced fever model, the ethanol extract notably reduced body temperature at both low (10 mg/kg) and medium (30 mg/kg) doses compared to the model group. Furthermore, both the petroleum ether and ethyl acetate fractions effectively lowered body temperature across all tested doses. The anti-pyretic effect of the ethyl acetate fraction at low (10 mg/kg) and medium (30 mg/kg) doses was particularly comparable to that of aspirin. The n-butanol fraction also demonstrated a significant anti-pyretic effect at the low dose (10 mg/kg). Additionally, all treatment groups of *V. yedoensis* extracts exhibited a reduction in total serum complement activity (CH_50_) levels, suggesting that these extracts may mitigate inflammatory responses by inhibiting complement system activity [17]. These observations indicate that extracts of *V. yedoensis*, particularly the petroleum ether and ethyl acetate fractions, possess notable anti-pyretic effects.

Another study investigated the influence of varying light intensities on the antipyretic activity of *V. yedoensis*. Shi et al. established a carrageenan-induced acute inflammation model and employed thermal infrared imaging and weighing techniques to assess local body temperature, heat radiation, and tissue swelling in mice. The results indicated that mice treated with *V. yedoensis* exhibited reductions in body temperature and swelling compared to the model group, with the effects intensifying with increased light intensity. Notably, *V. yedoensis* cultivated under full light conditions demonstrated the most pronounced anti-pyretic effect. Additionally, *V. yedoensis* significantly diminished succinate dehydrogenase (SDH) activity in the liver and lowered serum levels of inflammatory markers, including IL-1β, IL-6, TNF-α, and prostaglandin E_2_ (PGE_2_), with these effects correlating positively with light intensity. The most substantial inhibition of SDH activity was recorded in *V. yedoensis* grown under full light, suggesting that its anti-pyretic effects may be mediated through alterations in energy metabolism. Concurrently, the aqueous extract of *V. yedoensis* displayed an increased inhibition rate on A549 cells in response to rising light intensity, with samples grown under 100% light exhibiting significantly greater inhibition of A549 cell proliferation compared to other conditions [53]. These results indicate a close relationship between the anti-pyretic effects of *V. yedoensis* and the light intensity of its growing environment, thereby providing scientific rationale for optimizing its cultivation as an anti-pyretic herb.

### 5.3. Anti-Virus Effects

Viruses pose a significant threat to global human health, leading to a wide range of diseases and profoundly impacting the world economy [63,64,65]. *V. yedoensis*, a medicinal plant with a longstanding history, has traditionally been utilized for its detoxifying properties, which have been validated through clinical practice [66,67,68]. In modern medicine, the applications of *V. yedoensis* have expanded to encompass the effective prevention and treatment of various viral infections, highlighting its substantial traditional medical value. Furthermore, contemporary pharmacological studies affirm its role as a bridge between traditional and modern medicine. Early investigations identified potential anti-HIV active components within *V. yedoensis*. The crude extract demonstrates complete inhibition of Human Immunodeficiency Virus Type 1 (HIV-1) replication at sub-toxic concentrations, exhibiting an early inhibitory effect on viral replication influenced by the timing of application. Studies indicate that both the dimethyl sulfoxide (DMSO) extract and the methanol extract of *V. yedoensis* possess significant inhibitory effects on HIV-1 in vitro, although the anti-HIV-1 activity of the methanol extract is lower than that of the DMSO extract. Further research by Ngan et al. employed a range of separation techniques, including reverse-phase thin-layer chromatography, HPLC, and gel filtration chromatography, to successfully extract and purify a high molecular weight sulfated polysaccharide from the DMSO extract of *V. yedoensis*, which exhibited notable anti-HIV-1 activity in vitro. In antiviral activity assays, active components were co-cultured with HIV and H9 cells at various dilutions, with immunofluorescence techniques employed to stain and quantify HIV antigen-positive cells [54]. The results demonstrated that Fraction E of *V. yedoensis* displayed exceptional inhibitory effects on HIV-1, with a minimum inhibitory concentration (MIC) of 3 μg/mL, which is significantly lower than that of the unseparated extract (MIC > 200 μg/mL) and Fraction C (MIC = 50 μg/mL). At a concentration of 100 μg/mL, Fraction E exhibited antiviral characteristics comparable to those of sub-toxic untreated extracts; it did not induce interferon production, did not inactivate extracellular HIV or herpes simplex virus, and specifically inhibited HIV. Remarkably, this component retains its activity even at temperatures exceeding 100 °C. Additionally, cyclic peptides in *V. yedoensis*, particularly cycloviolacin Y5 (**144**), have demonstrated significant anti-HIV activity, with an EC_50_ value of 0.04 µM and an IC_50_ value of 1.8 µM, indicating robust antiviral properties. The study also established a positive correlation between the hydrophobicity of these cyclic peptides and their anti-HIV and hemolytic activities, suggesting that their effects may arise from interactions with cellular membranes [16]. Consequently, *V. yedoensis* is recognized as a rich source of potentially valuable anti-HIV compounds.

In a separate study, Liu et al. identified cyclic peptides from *V. yedoensis* that exhibit anti-H1N1 influenza virus activity. Notably, the newly discovered cyclic peptide, cycloviolacin VY1 (**162**), demonstrated remarkable anti-influenza virus properties in vitro, with an effective concentration range of 4.00 to 0.25 μg/mL, significantly lower than the positive control drug oseltamivir, which ranged from 156.25 to 9.77 μg/mL. Cycloviolacin VY1 achieved a maximum inhibition rate of 100% and a lower IC_50_ value of 2.27 ± 0.20 μg/mL, indicating greater potency compared to oseltamivir’s IC_50_ of 15.7 ± 0.80 μg/mL [50]. These data provide a robust scientific foundation for the development of antiviral drugs derived from *V. yedoensis*, offering new insights for future drug design and therapeutic strategies. Such results not only confirm the potential of *V. yedoensis* as an antiviral candidate but also lay a key groundwork for future drug development and antiviral approaches.

### 5.4. Anti-Cancer Effects

Cancer poses one of the most significant health challenges globally, with lung cancer exhibiting the highest mortality rate among all cancer types [69,70]. However, recent years have seen traditional therapies, particularly herbal medicine, offering new treatment options and renewed hope for many patients with lung cancer [71,72]. Increasing evidence suggests that extracts and active components of *V. yedoensis* possess anti-cancer properties. In a study by Huang et al., the effects of *V. yedoensis* extracts on the invasion capabilities of lung cancer cells, specifically human lung adenocarcinoma A549 cells and mouse Lewis lung cancer cells, were evaluated through in vitro experiments, along with an exploration of potential mechanisms of action. The results demonstrated that *V. yedoensis* extracts significantly reduced the invasiveness and migration of lung cancer cells at concentrations of 10, 25, 50, 75, and 100 µg/mL. This reduction was associated with the inhibition of matrix metalloproteinases (MMPs) and urokinase-type plasminogen activator (u-PA) activity, as well as the modulation of protease inhibitor levels, including TIMP-1, TIMP-2, and PAI-1. Furthermore, *V. yedoensis* extracts inhibited the DNA-binding capacity of NF-κB and influenced the p38 signaling pathway [55]. Collectively, these observations suggest that *V. yedoensis* extracts may inhibit lung cancer cell invasiveness by targeting the NF-κB signaling pathway and protease activity, providing valuable insights for the development of new anticancer strategies. Future research should aim to validate the anti-cancer effects and mechanisms of *V. yedoensis* using in vivo models.

### 5.5. Anti-Lung Injury Effects

Lung injury is primarily characterized by an inflammatory process that significantly damages the functionality of pulmonary capillary endothelial and alveolar epithelial cells [73,74]. Acute lung injury (ALI), however, extends beyond a localized inflammatory response; it is a severe condition with the potential to impact systemic health. This condition is especially prevalent among critically ill patients and is associated with high morbidity and mortality rates [75,76,77]. Empirical evidence suggests that the lipid-soluble extract of *V. yedoensis* may exhibit beneficial effects against ALI [78]. Li et al. investigated the therapeutic potential of this extract in a mouse model induced by LPS, effectively simulating the pathological state of human ALI. Results demonstrated that the lipid-soluble extract of *V. yedoensis* significantly reduced pulmonary edema at doses of 2, 4, and 8 mg/kg, as indicated by a marked decrease in the lung wet/dry weight ratio. Additionally, the extract modulated cell counts and protein levels in bronchoalveolar lavage fluid, diminishing inflammation-induced cellular infiltration and vascular permeability. It further reduced the levels of key pro-inflammatory cytokines, including TNF-α, IL-1β, and IL-6, which are pivotal in the inflammatory cascade. Histological evaluations corroborated the protective effects on alveolar tissue, showing reduced thickening of the alveolar wall and lower inflammatory cell accumulation. Notably, the lipid-soluble extract decreased LPS-induced pulmonary complement deposition, indicating its potential to inhibit inflammatory responses through modulation of the complement system [56]. Collectively, these results illustrate that the lipid-soluble extract of *V. yedoensis* provides significant protection against ALI by inhibiting excessive complement activation and reducing pro-inflammatory cytokine production. Thus, *V. yedoensis* not only establishes a scientific foundation for ALI treatment but also paves the way for future drug development and clinical applications.

### 5.6. Anti-Liver Injury Effects

The liver is particularly vulnerable to injury in cases of abdominal trauma, with increased severity and grading of liver damage significantly heightening the risks of hemorrhage and mortality, thereby posing a long-term threat to global health [79,80]. Despite remarkable advancements in modern medicine, safe and effective pharmacological treatments for liver injuries remain elusive, constituting a major challenge within the medical field. In TCM, *V. yedoensis* is associated with the liver meridian and is believed to have hepatoprotective effects [15]. Recent studies have underscored the promising in vitro and in vivo protective effects of *V. yedoensis* extract against immune-mediated liver injury (ILI). Reports indicate that the n-butanol extract of *V. yedoensis* exhibits potential in addressing ILI. Chu et al. systematically evaluated the protective efficacy of this extract. In their in vivo experiments, a ConA-induced mouse model was employed to simulate ILI, with mice receiving oral doses of 3.00, 6.00, and 12.00 g/kg/day for seven consecutive days. Diammonium glycyrrhizinate served as a positive control at a dose of 100 mg/kg. Serum samples were collected to measure ALT and AST levels, while ELISA was used to assess GSH-PX, superoxide dismutase (SOD), malondialdehyde (MDA), and the inflammatory factors TNF-α and IFN-γ in the liver. Histopathological examinations were also conducted to evaluate liver damage. In vitro, the HepG2.2.15 cell line, containing HBV DNA, was utilized to model a hepatocyte environment for HBV infection. Various concentrations of the n-butanol extract (ranging from 0.0001 to 1.0 mg/mL) were tested, with Lamivudine (3TC) at 0.05 mg/mL serving as a positive control. In vivo findings revealed that the n-butanol extract of *V. yedoensis* significantly reduced serum levels of ALT, AST, and MDA while enhancing the activities of SOD and GSH-PX. Additionally, the extract inhibited the production of pro-inflammatory factors TNF-α and IFN-γ, resulting in improved pathological liver conditions. In vitro experiments demonstrated that the extract effectively suppressed the secretion of HBsAg and HBeAg, as well as HBV DNA replication [15]. Furthermore, various active compounds were isolated and characterized from the n-butanol extract using silica gel column chromatography and mass spectrometry. These compounds included coumarin derivatives (such as esculetin (**41**), prionanthoside (**56**), cichoriin (**57**), and esculin (**58**)) and flavonoids (such as quercetin-3-*O*-galactoside (**18**)), providing a chemical foundation for the hepatoprotective effects of *V. yedoensis*. In summary, the n-butanol extract of *V. yedoensis* exhibits significant protective effects against immune-mediated liver injury through its antioxidant and anti-inflammatory mechanisms, offering promising insights for future drug development.

### 5.7. Anti-Bacterial Effects

Previous research has confirmed the antibacterial activity of crude extracts from *V. yedoensis* [3]. Xie et al. demonstrated that the petroleum ether and ethyl acetate extracts exhibited inhibitory effects against *Bacillus subtilis* and *Pseudomonas syringae*, whereas methanol and methanol–water extracts showed no antibacterial activity. Further investigation revealed that the petroleum ether extracts significantly inhibited *B. subtilis* at a concentration of 6.25 µg/mL. Coumarin compounds, recognized as key bioactive constituents of *V. yedoensis*, are particularly noted for their antibacterial properties. Pharmacological studies have extensively documented the antibacterial characteristics of coumarins found in this plant. Notably, four coumarin compounds with significant antibacterial activity were isolated through methanol extraction and advanced chromatographic techniques: esculetin (**41**), 6,7-dimethoxycoumarin (**45**), scopoletin (**54**), and the newly identified compound 5-methoxy-7-hydroxymethylcoumarin (**65**). These coumarin derivatives exhibit varying degrees of inhibitory and bactericidal effects against a range of animal pathogens, including *Staphylococcus aureus*, *Escherichia coli*, *Streptococcus lactis*, *Streptococcus agalactiae*, *Streptococcus dysgalactiae*, and *Salmonella*. Among these, esculetin demonstrates the most potent antibacterial efficacy, with MIC values ranging from 0.031 to 0.313 g/L and minimum bactericidal concentration (MBC) values between 0.313 and 0.625 g/L [32]. In summary, *V. yedoensis* exhibits significant antibacterial effects against various animal pathogenic bacteria, indicating that coumarin compounds are likely the primary agents responsible for its antimicrobial activity. These insights establish a scientific foundation for the potential applications of *V. yedoensis* in veterinary medicine and underscore the necessity for further research on the coumarin compounds present in this plant.

### 5.8. Other Effects

In addition to the aforementioned pharmacological effects, *V. yedoensis* exhibits other significant actions. In the context of the blood system, it demonstrates noteworthy anticoagulant properties. Zhou et al. conducted in vitro experiments to evaluate the anticoagulant activity of coumarin compounds derived from *V. yedoensis*. Utilizing silica gel column chromatography, Sephadex LH-20 chromatography, and recrystallization techniques, they isolated a novel bis-coumarin, dimeresculetin (**40**), along with two known compounds, esculetin (**41**) and euphorbetin (**42**). The results indicated that these coumarin compounds, at concentrations of 25, 60, and 100 µg/mL, significantly influenced activated partial thromboplastin time (APTT), thrombin time (TT), and prothrombin time (PT). Notably, at 100 µg/mL, dimeresculetin extended TT to 74.97 s, slightly surpassing the positive control heparin (73.16 s). However, dimeresculetin did not significantly affect APTT, suggesting its primary action on the extrinsic coagulation pathway. In contrast, euphorbetin and esculetin exhibited substantial anticoagulant activity across APTT, PT, and TT, with their effects exceeding those of dimeresculetin at certain concentrations [1]. Collectively, the bis-coumarin compounds from *V. yedoensis* demonstrate significant anticoagulant properties by targeting distinct coagulation pathways, making them promising candidates for the development of novel antithrombotic agents.

Furthermore, sesquiterpenes and alkaloids identified in *V. yedoensis* have gained attention for their potential anti-complement activity. Du et al. successfully isolated and characterized two novel germacrane-type sesquiterpenes, yedoensins A (**66**) and B (**67**), along with several known compounds. Functional validation revealed that compounds 1–3 and 5–9 exhibited significant anti-complement activity, with CH_50_ and AP_50_ values targeting both the classical pathway (CP) and the alternative pathway (AP) ranging from 0.14 to 0.37 mg/mL and 0.32 to 0.54 mg/mL, respectively. Preliminary mechanistic studies using complement-depleted serum indicated that compounds 1 and 3 interact with C1q, C3, and C9, while compounds 6, 7, and 9 target C1q, C3, C5, and C9, suggesting their involvement in the complement activation cascade. Additionally, Du et al. identified novel alkaloids from the 95% ethanol extract of *V. yedoensis*, which were confirmed to possess anti-complement activity. These compounds were evaluated in vitro for their effects on both the classical and alternative pathways of the complement system, with most exhibiting significant anti-complement activity, demonstrating CH_50_ and AP_50_ values in the range of 0.12 to 0.50 g/L [31].

Moreover, *V. yedoensis* serves as a feed additive that significantly enhances the activity of antioxidant enzymes, such as catalase (CAT) and SOD, reduces MDA production, and decreases the number of apoptotic cells, as well as the Bax/Bcl-2 ratio. Furthermore, *V. yedoensis* effectively mitigates LPS-induced oxidative stress in the intestines by modulating the NF-κB-NLRP3 and Nrf2-MAPK signaling pathways, providing protective effects against intestinal inflammation and oxidative damage.

## 6. Quality Control

As biologically active natural products, quality control in TCM is fundamental to ensuring clinical efficacy. Accurate identification of medicinal materials constitutes a core element of TCM quality control, as substituting similar or unrelated species can adversely affect therapeutic outcomes. The genus *Viola* comprises over 500 species worldwide, distributed across tropical, subtropical, and temperate regions [11]. In China, approximately 117 species of *Viola* have been documented, most of which exhibit significant biological activity and medicinal value. Notably, many *Viola* species, such as *V. odorata*, *V. tianshanica*, and *V. mandshurica*, share high morphological similarities with *V. yedoensis*, leading to potential confusion. Misidentified or adulterated species may exhibit pharmacological effects that diverge considerably from those of *V. yedoensis*. For example, *Viola betonicifolia* J. E. Smith has been shown to promote intestinal motility and induce diarrhea. Incorrectly identifying *Viola betonicifolia* as *V. yedoensis* poses significant safety risks [81,82]. Therefore, developing analytical methods to effectively distinguish *V. yedoensis* from related species is urgently needed. Recent advancements in identification techniques for *V. yedoensis* have emerged, including ultraviolet spectroscopy (UV), HPLC, liquid chromatography–mass spectrometry (LC-MS), and Fourier-transform infrared spectroscopy (FT-IR) [83,84,85]. These techniques provide valuable means of assessing the quality of *V. yedoensis*. Representative methods for quality control encompass single-component quantification, multi-component content analysis, and fingerprinting. The primary quantitative components in these analyses are flavonoids and coumarins, attributed to their high concentrations in *V. yedoensis*.

ChP, 2020 designates *Viola yedoensis* Makino as the sole plant source of *V. yedoensis*. According to the pharmacopoeia, quality control measures for *V. yedoensis* encompass the identification of the medicinal material, thin-layer chromatography (TLC) analysis, moisture content assessment, total ash determination, detection of acid-insoluble ash, and quantification of extracts. Notably, the concept of the quality marker (Q-marker) represents a novel approach to TCM quality control. The ChP, 2020 identifies esculetin as the Q-marker for *V. yedoensis*, stipulating that its content must not be less than 0.20% when calculated on a dry weight basis. However, given the multi-component and multi-target nature of TCM, relying exclusively on esculetin content is insufficient for evaluating the quality of *Viola violacea*. Furthermore, esculetin is also found in certain counterfeit products, indicating that current pharmacopoeia standards may not effectively identify adulterated *V. yedoensis*. Consequently, there is an urgent need to develop a simple, rapid, accurate, and reliable quality control method to ensure the integrity of *V. yedoensis*. Recent years have witnessed the integration of various techniques, including HPLC, ultra-high-performance liquid chromatography–tandem mass spectrometry (UHPLC-MS), and ESI-MS, for the qualitative analysis of *V. yedoensis*. A recent study by Chen et al. utilized ultra-high-performance liquid chromatography–quadrupole–electrostatic field orbitrap tandem mass spectrometry (UHPLC-Q-Orbitrap-MS/MS) to establish identification and semi-quantification methods for 36 compounds in *V. yedoensis*. This method successfully identified multiple compounds, distinguishing between four isomers: vitexin and iso-vitexin, as well as schaftoside and iso-schaftoside. Semi-quantitative analysis revealed that the chemical content of these 36 compounds ranged from 0.001% to 1.958%, with esculetin measured at 0.484 ± 0.028%. Additionally, the study proposed quercetin as a new supplementary Q-marker for *V. yedoensis*, enhancing the accuracy of its identification [86]. Together with esculetin, this new Q-marker system could more effectively differentiate genuine *V. yedoensis* from commercial counterfeits and inferior products.

In a separate study, high-performance liquid chromatography–electrospray ionization–ion trap tandem mass spectrometry (HPLC-ESI-IT-MS^n^) facilitated a comprehensive qualitative analysis of C-glycoside flavonoids in *V. yedoensis*, alongside a detailed investigation of their fragmentation mechanisms. This advancement offers a novel analytical strategy for the quality control of *V. yedoensis*. The use of negative mode electrospray ionization enabled effective differentiation and identification of C-glycosylation types among flavonoids, as well as their distribution at the C-6 and C-8 positions. Moreover, the application of MS^n^ technology elucidated the types of sugar units (e.g., hexose or pentose) and their fragmentation patterns. The implementation of HPLC-MS^n^ significantly enhanced the accuracy of identifying structural types of compounds in *V. yedoensis*, particularly for complex or difficult-to-distinguish substances [87]. Zhang et al. successfully established a rapid and sensitive analytical method employing high-performance liquid chromatography–diode array detection–electrospray ionization mass spectrometry (HPLC-DAD-ESI-MS) to isolate and identify anthocyanins from *V. yedoensis*. This method not only improved the efficiency of anthocyanin identification but also introduced a new strategy for quality control. It enabled the analysis of 14 types of anthocyanins, including non-acylated glycosides, acetylated glycosides, and coumaroyl glycosides, within 40 min, thus providing a scientific basis for the standardization and quality assessment of *V. yedoensis* [88]. Notably, significant differences in anthocyanin composition at the aglycone level and acylation patterns were observed between *V. yedoensis* and its closely related species, *V. prionantha*.

Additionally, chemical fingerprinting serves as a comprehensive and specific method for the quality control of *V. yedoensis*. The HPLC-UV fingerprinting technique provides a quantitative approach to identifying bioactive components, including the flavonoid quercetin-3-*O*-β-d-glucoside and coumarins such as cichoriin, esculetin, scopoletin, prionanthoside, and euphorbetin. These components exhibit strong linear relationships within their respective calibration ranges (r > 0.9996), indicating that this method possesses accuracy, stability, and good reproducibility, making it suitable for the quality control of *V. yedoensis*. Furthermore, principal component analysis and cluster analysis enable precise quality assessments and differentiation of *V. yedoensis* from related species [89]. The integration of chemometric techniques with HPLC has proven to be a sensitive, selective, rapid, and accurate method for quality evaluation.

Notably, relying solely on the evaluation of individual compounds or a limited number of compounds is an unreliable approach for quality assessment. *V. yedoensis* contains a diverse array of phytochemical structures, some of which are challenging to extract and purify or exist only in trace amounts, presenting obstacles to quality control. Currently, research on the quality control of *V. yedoensis* remains in its early stages, lacking standardized indicator components and testing criteria. Additionally, shortcomings in detection parameters, such as the unclear composition of common peaks in its fingerprinting profile, necessitate further investigation to optimize quality assessment methods. Therefore, building on existing research and enhancing quality assessment standards through multiple methods, indicators, and dimensions is essential to ensure the safety, reliability, and efficacy of *V. yedoensis* in clinical applications.

## 7. Potential and Actual Applications

**Figure 8:**
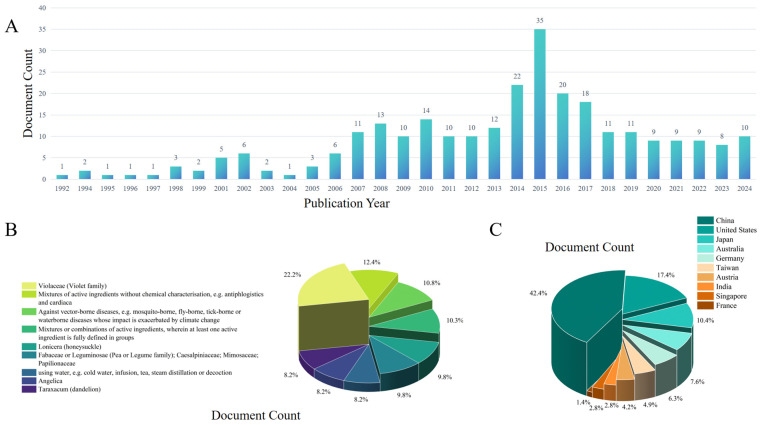
Current situation of patent inventions related to *V. yedoensis*. (**A**) Document numbers, (**B**) application of patents, (**C**) patent distribution.

*V. yedoensis* possesses significant medicinal value in TCM and demonstrates distinctive application potential in various fields, including food, pharmaceuticals, cosmetics, and animal husbandry. Recent statistics from patent databases indicate that a total of 275 patents related to *V. yedoensis* have been registered globally, with the majority concentrated in the food and pharmaceutical sectors (https://www.lens.org/, Figure 8). As the demand for natural health products continues to rise, research and development efforts surrounding *V. yedoensis* and associated patent applications are anticipated to gain increased attention. In the food industry, herbal tea derived from *V. yedoensis* extract is recognized for its heat-clearing and detoxifying properties, potentially mitigating inflammation. Furthermore, the extract is utilized in the formulation of functional foods and nutritional supplements [90]. Scientific investigations have identified various minerals and trace elements in *V. yedoensis* that are beneficial to human health, including sodium, calcium, magnesium, aluminum, silicon, chlorine, and iron, all of which are essential for maintaining normal physiological functions [91]. Within the pharmaceutical sector, *V. yedoensis* serves as an indispensable ingredient in TCM formulations. It is employed in various dosage forms, including oral solutions and tablets, commonly used to treat conditions such as pharyngitis, the common cold, and skin infections [92]. Topical ointments containing *V. yedoensis* extract have demonstrated effectiveness in alleviating symptoms associated with skin inflammation, eczema, and ulcers. Additionally, its active compounds exhibit potential antitumor properties, suggesting possibilities for developing it into a natural anticancer drug or adjunct therapy in the future. In the cosmetics industry, the anti-inflammatory properties of *V. yedoensis* are leveraged in serums designed to reduce skin irritation and swelling [93]. Moreover, its extract is incorporated into antioxidant creams aimed at slowing the aging process and protecting the skin from oxidative damage. In animal husbandry, *V. yedoensis* is increasingly recognized as a natural feed additive. Studies have shown that its anti-inflammatory and antioxidant properties, when used as a dietary supplement, can protect broiler chickens from intestinal inflammation and oxidative damage.

In addition to the aforementioned areas, *V. yedoensis* also demonstrates potential applications in environmental protection and agriculture. As a phytoremediation agent, it is utilized to restore soils contaminated with heavy metals, particularly in areas impacted by cadmium (Cd) pollution [94]. Its adaptability to Cd-contaminated environments offers a unique perspective for studying the mechanisms underlying heavy metal tolerance. In a recent study, Peng et al. employed transcriptome sequencing technology to investigate the gene expression response of *V. yedoensis* under Cd stress. They successfully developed new molecular markers, including simple sequence repeats (SSRs) and single nucleotide polymorphisms (SNPs). The study generated approximately 91.99 million high-quality clean reads and predicted 11,176 SSRs, which were distributed across 9644 unigenes, with 1283 unigenes containing multiple SSRs. Additionally, the study identified 8250 and 11,328 SNP markers in the VIYCd and VIYCK libraries, respectively. The development of these SSR and SNP markers not only provides valuable resources for the genetic analysis of *V. yedoensis* but also lays a foundation for further research in its genomics and functional genomics. Through precise gene editing, it is possible to target and regulate genes associated with the synthesis of secondary metabolites, thereby enhancing the production of medicinally valuable phytochemicals or other bioactive compounds in *V. yedoensis*. For example, genes from the cyclopeptide family in *V. yedoensis* have been shown to be closely associated with defense responses and DNA repair processes. Editing these genes is expected to enhance the plant’s resistance to stress while promoting the accumulation of its medicinal components. Moreover, genomic editing can also optimize the plant’s growth and developmental traits, making it more adaptable to various environmental conditions and thereby increasing its potential in agricultural production and medicinal plant cultivation [95]. In summary, these findings not only improve our understanding of the cadmium tolerance mechanisms in *V. yedoensis* but also open new possibilities for enhancing its medicinal value through gene editing technologies.

## 8. Conclusions and Prospects

This paper reviews the research progress on *V. yedoensis* in various domains, including botany, traditional uses, phytochemistry, pharmacology, quality control, and potential applications. *V. yedoensis* has a long-standing history of medicinal use in China and shows significant promise, particularly in anti-inflammatory therapies. Phytochemical analyses have identified numerous active compounds within *V. yedoensis*, including flavonoids, coumarins, terpenoids, alkaloids, phenolic acids, and cyclopeptides. Previous studies predominantly highlight flavonoids and coumarins as the primary bioactive constituents. Modern pharmacological research, through various in vitro and in vivo experiments, has confirmed the remarkable health benefits of this plant. Extensive studies indicate that extracts of *V. yedoensis* possess significant anti-inflammatory, anti-pyretic, anti-viral, anti-cancer, anti-lung injury, anti-liver injury, anti-bacterial, anti-coagulant, and anti-oxidant activities. These findings substantiate many of the traditional applications of the plant in TCM. Additionally, this review underscores the potential value and practical applications of *V. yedoensis* across the food, pharmaceutical, cosmetic, and animal husbandry sectors, providing new insights for its future development.

Despite the extensive research on *V. yedoensis*, certain shortcomings persist. First, current literature predominantly identifies flavonoids and coumarins as the primary chemical constituents, while investigations into other chemical components remain relatively sparse. To elucidate the relationship between bioactive compounds and pharmacological effects, it is crucial to employ advanced technologies to isolate and identify the diverse secondary metabolites present in the plant, including alkaloids, terpenoids, and polysaccharides. These compounds may play critical roles in pharmacological activities but have not received adequate attention in existing research. Techniques such as liquid chromatography–tandem mass spectrometry (LC-MS/MS) and NMR can be applied for a systematic analysis of these compounds’ structures. Additionally, omics approaches, such as metabolomics, can be utilized to explore the synergistic effects of these compounds across various pharmacological activities. This comprehensive approach would offer deeper insights into the potential mechanisms linking the chemical composition of *V. yedoensis* to its biological activities.

Second, current research on *V. yedoensis* extraction primarily emphasizes crude extracts, resulting in insufficient exploration of its precise chemical basis. Historically, *V. yedoensis* has been a valuable resource in TCM for treating inflammation-related diseases, and modern medical applications have demonstrated its excellent efficacy in anti-inflammatory treatments. The primary mechanism involves significantly reducing levels of inflammatory cytokines such as IL-1β, TNF-α, and IL-6, particularly through the modulation of key pathways like NF-κB-NLRP3 and Nrf2-MAPK. Although pharmacological studies have largely focused on crude extracts, investigations into individual compounds remain limited. This gap is noteworthy, as individual compounds are critical for new drug development. Consequently, a deeper investigation into the bioactive compounds of *V. yedoensis* is essential for future research. While numerous studies highlight the plant’s diverse biological activities, most have been confined to in vitro or animal models, potentially failing to capture the complexities involved in preclinical or clinical studies. Therefore, further research is urgently needed to bridge these gaps and advance the understanding of *V. yedoensis*’s therapeutic potential.

Third, quality control studies of *V. yedoensis* predominantly focus on evaluating individual compounds or a limited number of components. Currently, esculetin serves as a marker substance for assessing quality; however, reliance on a single compound is inadequate to fully represent the pharmacological profile or ensure safety. Future research should integrate fingerprint analysis with network pharmacology to predict comprehensive quality markers for *V. yedoensis*. This approach would enhance the understanding of the plant’s overall quality characteristics and support more diverse quality control strategies, ultimately leading to a more robust evaluation system.

Fourth, research on the pharmacokinetics of *V. yedoensis* remains scarce, with unclear pharmacokinetic data for its key active compounds and extracts. Pharmacokinetic studies are essential for minimizing adverse drug reactions, enhancing therapeutic efficacy, and providing valuable guidance for clinical use. Strengthening research on the pharmacokinetics of *V. yedoensis* is crucial for a better understanding of the absorption, distribution, metabolism, and excretion of its constituents in the body.

Overall, *V. yedoensis* is a valuable resource for promoting human health. This study presents the first comprehensive review of *V. yedoensis*, aiming to provide meaningful insights and references to guide future research and development efforts.

## Figures and Tables

**Table 1:** Chemical compounds isolated from *V. yedoensis*.

No.	Compounds	Molecular Formula	Identification Method	Extraction Method	Extraction Solvent	Isolation and Purification Procedure	References
Flavonoids							
**1**	Apigenin-6-*C*-α-l-arabinopyranosyl-8-*C*-β-l-arabinopyranoside	C_25_H_26_O_13_	NMR, DQF-COSY, HSQC, HMBC	Sequential solvent extraction	Methanol	Amberlite XAD2 column chromatography, Sephadex LH-20 column chromatography, Semi-preparative HPLC	[4]
**2**	Apigenin 6,8-di-*C*-α-l-arabinopyranoside	C_25_H_26_O_13_	NMR, DQF-COSY, HSQC, HMBC	Sequential solvent extraction	Methanol	Amberlite XAD2 column chromatography, Sephadex LH-20 column chromatography, Semi-preparative HPLC	[4]
**3**	Schaftoside	C_26_H_28_O_14_	NMR, DQF-COSY, HSQC, HMBC	Sequential solvent extraction	Methanol	Amberlite XAD2 column chromatography, Sephadex LH-20 column chromatography, Semi-preparative HPLC	[4]
**4**	Neoschaftoside	C_26_H_28_O_14_	NMR, DQF-COSY, HSQC, HMBC	Sequential solvent extraction	Methanol	Amberlite XAD2 column chromatography, Sephadex LH-20 column chromatography, Semi-preparative HPLC	[4]
**5**	Apigenin-6-*C*-α-l-arabinopyranosyl-8-*C*-β-d-xylopyranoside	C_25_H_26_O_13_	NMR, DQF-COSY, HSQC, HMBC	Sequential solvent extraction	Methanol	Amberlite XAD2 column chromatography, Sephadex LH-20 column chromatography, Semi-preparative HPLC	[4]
**6**	Apigenin-6-*C*-β-d-xylopyranosyl-8-*C*-α-l-arabinopyranoside	C_25_H_26_O_13_	NMR, DQF-COSY, HSQC, HMBC	Sequential solvent extraction	Methanol	Amberlite XAD2 column chromatography, Sephadex LH-20 column chromatography, Semi-preparative HPLC	[4]
**7**	Isocarlinoside	C_26_H_28_O_15_	NMR, DQF-COSY, HSQC, HMBC	Sequential solvent extraction	Methanol	Amberlite XAD2 column chromatography, Sephadex LH-20 column chromatography, Semi-preparative HPLC	[4]
**8**	Vicenin-2	C_27_H_30_O_15_	NMR, DQF-COSY, HSQC, HMBC	Sequential solvent extraction	Methanol	Amberlite XAD2 column chromatography, Sephadex LH-20 column chromatography, Semi-preparative HPLC	[4,5]
**9**	Isoorientin	C_21_H_20_O_11_	NMR, DQF-COSY, HSQC, HMBC	Sequential solvent extraction	Methanol	Amberlite XAD2 column chromatography, Sephadex LH-20 column chromatography, Semi-preparative HPLC	[4,5]
**10**	Apigenin-6,8-di-*C*-α-l-arabinopyranoside	C_25_H_26_O_13_	NMR, DQF-COSY, HSQC, HMBC	Sequential solvent extraction	Methanol	Amberlite XAD2 column chromatography, Sephadex LH-20 column chromatography, Semi-preparative HPLC	[4,13]
**11**	Isoschaftoside	C_26_H_28_O_14_	NMR, DQF-COSY, HSQC, HMBC	Sequential solvent extraction	Methanol	Amberlite XAD2 column chromatography, Sephadex LH-20 column chromatography, Semi-preparative HPLC	[4,13]
**12**	Isovitexin	C_21_H_20_O_10_	NMR, HPLC, MALDI-TOF MS	Sequential solvent extraction	50% Ethanol	Silica gel column chromatography, Activated carbon column chromatography, Diaion HP-20 column chromatography	[5]
**13**	Isoscoparin	C_22_H_22_O_11_	NMR, HPLC, MALDI-TOF MS	Sequential solvent extraction	50% Ethanol	Silica gel column chromatography, Activated carbon column chromatography, Diaion HP-20 column chromatography	[5]
**14**	Kaempferol-3-*O*-β-d-Glucosyl-7-*O*-α-l-rhamnosylkaempferol	C_27_H_34_O_17_	NMR, HPLC, MALDI-TOF MS	Sequential solvent extraction	50% Ethanol	Silica gel column chromatography, Activated carbon column chromatography, Diaion HP-20 column chromatography	[5]
**15**	Rutin	C_27_H_30_O_16_	NMR, HPLC, MALDI-TOF MS	Sequential solvent extraction	50% Ethanol	Silica gel column chromatography, Activated carbon column chromatography, Diaion HP-20 column chromatography	[5,22]
**16**	5-Hydroxy-4′-methoxyflavone-7-*O*-rutinoside	C_28_H_32_O_14_	NMR, MS, CD spectroscopic data	N/A	N/A	Silica gel column chromatography, Thin-layer chromatography, Recrystallization	[6]
**17**	Apigenin-6-*C*-β-d-glucopyranosyl-8-*C*-α-l-arabinopyranoside	C_26_H_28_O_14_	NMR, MS, CD spectroscopic data	N/A	N/A	Silica gel column chromatography, Thin-layer chromatography, Recrystallization	[6]
**18**	Quercetin-3-*O*-galactoside	C_21_H_20_O_12_	NMR, MS	Ultrasonic extraction	60% Ethanol	Silica gel column chromatography, Recrystallization	[15]
**19**	Diosmetin	C_16_H_12_O_6_	UHPLC-Q-Orbitrap-MS	Reflux extraction method	75% Ethanol	N/A	[22]
**20**	Morin	C_15_H_10_O_7_	UHPLC-Q-Orbitrap-MS	Reflux extraction method	75% Ethanol	N/A	[22]
**21**	Astragalin	C_21_H_20_O_11_	UHPLC-Q-Orbitrap-MS	Reflux extraction method	75% Ethanol	N/A	[22]
**22**	Quercitrin	C_21_H_20_O_11_	UHPLC-Q-Orbitrap-MS	Reflux extraction method	75% Ethanol	N/A	[22]
**23**	1,5-Anhydro-1-[2-(3,4-dihydroxyphenyl)-5,7-dihydroxy-4-oxo-4*H*-chromen-8-yl]-d-galactitol	C_21_H_20_O_11_	UHPLC-Q-Orbitrap-MS	Reflux extraction method	75% Ethanol	N/A	[22]
**24**	Quercetin-3-β-d-glucoside	C_21_H_20_O_12_	UHPLC-Q-Orbitrap-MS	Reflux extraction method	75% Ethanol	N/A	[22]
**25**	Quercetin	C_15_H_10_O_7_	UHPLC-Q-Orbitrap-MS	Reflux extraction method	75% Ethanol	N/A	[22]
**26**	Isorhamnetin	C_16_H_12_O_7_	UHPLC-Q-Orbitrap-MS	Reflux extraction method	75% Ethanol	N/A	[22]
**27**	Quercetin-3-*O*-β-d-glucoside	C_21_H_20_O_12_	NMR, ESI-MS	Solvent extraction method	75% Ethanol	Silica gel column chromatography	[23]
**28**	Kaempferol-3-*O*-β-d-glucoside	C_21_H_20_O_9_	NMR, ESI-MS	Solvent extraction method	75% Ethanol	Silica gel column chromatography	[23]
**29**	Apigenin	C_15_H_10_O_5_	NMR, ESI-MS	Solvent extraction method	75% Ethanol	Silica gel column chromatography	[23]
**30**	Techtochrysin	C_16_H_12_O_4_	HPLC, NMR, MS	Reflux extraction method	95% Ethanol	Silica gel column chromatography, ODS column chromatography, Sephadex LH-20 gel column chromatography, Preparative high-performance liquid chromatography	[24]
**31**	Naringenin	C_15_H_12_O_5_	NMR, MS	Reflux extraction method	Ethyl acetate	Silica gel column chromatography	[25]
**32**	Luteolin	C_15_H_10_O_6_	NMR, MS	Reflux extraction method	Ethyl acetate	Silica gel column chromatography	[25]
**33**	Keampferol-3-*O*-β-d-glucosyl-(1-2)-*O*-α-l-rhamnoside	C_27_H_30_O_15_	NMR, MS	Reflux extraction method	Ethyl acetate	Silica gel column chromatography	[25]
**34**	Quercetin-3-*O*-β-d-glucosyl-(1-4)-*O*-α-l-rhamnoside	C_27_H_30_O_16_	NMR, MS	Reflux extraction method	Ethyl acetate	Silica gel column chromatography	[25]
**35**	Chrysoeriol	C_16_H_12_O_6_	HPLC, MS, NMR	Solvent extraction method	95% Ethanol	Macroporous resin adsorption	[26]
**36**	Acacetin-7-*O*-β-d-glucoside	C_22_H_22_O_10_	HPLC, MS, NMR	Solvent extraction method	95% Ethanol	Macroporous resin adsorption	[26]
**37**	Acacetin-7-*O*-β-d-apiosyl-(1-2)-β-d-glucoside	C_27_H_30_O_14_	HPLC, MS, NMR	Solvent extraction method	95% Ethanol	Macroporous resin adsorption	[26]
**38**	Vicenin-3	C_26_H_28_O_14_	NMR, MS	Solvent extraction method	N/A	N/A	[27]
**39**	Kaempferol-3-*O*-β-d-sophorose-7-*O*-α-l-rhamnoside	C_33_H_40_O_20_	NMR, MS	Solvent extraction method	N/A	N/A	[27]
Coumarins							
**40**	Dimeresculetin	C_18_H_10_O_8_	NMR, HSQC, HMBC, ROEST, UV, IR	Reflux extraction method	Ethyl acetate	Silica gel column chromatography	[1]
**41**	Esculetin	C_9_H_6_O_4_	NMR, HSQC, HMBC, ROEST, UV, IR	Reflux extraction method	Ethyl acetate	Silica gel column chromatography	[1,5,22,28]
**42**	Euphorbetin	C_18_H_10_O_8_	NMR, HSQC, HMBC, ROEST, UV, IR	Reflux extraction method	Ethyl acetate	Silica gel column chromatography	[1,28]
**43**	7-Hydroxycoumarine	C_9_H_6_O_3_	NMR, HPLC, MALDI-TOF MS	Sequential solvent extraction	50% Ethanol	Silica gel column chromatography, Activated carbon column chromatography, Diaion HP-20 column chromatography	[5]
**44**	6-Hydroxy-7-methoxycoumarin	C_10_H_8_O_4_	NMR, HPLC, MALDI-TOF MS	Sequential solvent extraction	50% Ethanol	Silica gel column chromatography, Activated carbon column chromatography, Diaion HP-20 column chromatography	[5]
**45**	6,7-Dimethoxycoumarin	C_11_H_10_O_4_	NMR, HPLC, MALDI-TOF MS	Sequential solvent extraction	50% Ethanol	Silica gel column chromatography, Activated carbon column chromatography, Diaion HP-20 column chromatography	[5]
**46**	6-Hydroxy-7-ethoxycoumarin	C_11_H_10_O_4_	NMR, HPLC, MALDI-TOF MS	Sequential solvent extraction	50% Ethanol	Silica gel column chromatography, Activated carbon column chromatography, Diaion HP-20 column chromatography	[5]
**47**	6,7-Dibutoxycoumarin	C_17_H_22_O_4_	NMR, HPLC, MALDI-TOF MS	Sequential solvent extraction	50% Ethanol	Silica gel column chromatography, Activated carbon column chromatography, Diaion HP-20 column chromatography	[5]
**48**	6-Hydroxy-7-butoxycoumarin	C_13_H_14_O_4_	NMR, HPLC, MALDI-TOF MS	Sequential solvent extraction	50% Ethanol	Silica gel column chromatography, Activated carbon column chromatography, Diaion HP-20 column chromatography	[5]
**49**	6,7-Dioctyloxycoumarin	C_25_H_38_O_4_	NMR, HPLC, MALDI-TOF MS	Sequential solvent extraction	50% Ethanol	Silica gel column chromatography, Activated carbon column chromatography, Diaion HP-20 column chromatography	[5]
**50**	6-Hydroxy-7-octyloxycoumarin	C_17_H_22_O_4_	NMR, HPLC, MALDI-TOF MS	Sequential solvent extraction	50% Ethanol	Silica gel column chromatography, Activated carbon column chromatography, Diaion HP-20 column chromatography	[5]
**51**	2,3-Dihydro-7*H*-pyrano[2,3-g]-1,4-benzodioxin-7-one	C_11_H_8_O_4_	NMR, HPLC, MALDI-TOF MS	Sequential solvent extraction	50% Ethanol	Silica gel column chromatography, Activated carbon column chromatography, Diaion HP-20 column chromatography	[5]
**52**	6-Ethoxy-7-methoxycoumarin	C_12_H_12_O_4_	NMR, HPLC, MALDI-TOF MS	Sequential solvent extraction	50% Ethanol	Silica gel column chromatography, Activated carbon column chromatography, Diaion HP-20 column chromatography	[5]
**53**	7-Ethoxy-6-methoxycoumarin	C_12_H_12_O_4_	NMR, HPLC, MALDI-TOF MS	Sequential solvent extraction	50% Ethanol	Silica gel column chromatography, Activated carbon column chromatography, Diaion HP-20 column chromatography	[5]
**54**	Scopoletin	C_10_H_8_O_4_	NMR, HPLC, MALDI-TOF MS	Sequential solvent extraction	50% Ethanol	Silica gel column chromatography, Activated carbon column chromatography, Diaion HP-20 column chromatography	[5,22]
**55**	6,6’,7,7’-Tetrahydroxy-5,8’-biscoumarin	C_18_H_10_O_8_	NMR, MS, CD spectroscopic data	N/A	N/A	Silica gel column chromatography, Thin-layer chromatography, Recrystallization	[6]
**56**	Prionanthoside	C_17_H_18_O_10_	NMR, MS	Ultrasonic extraction	60% Ethanol	Silica gel column chromatography, Recrystallization	[15]
**57**	Cichoriin	C_15_H_16_O_9_	NMR, MS	Ultrasonic extraction	60% Ethanol	Silica gel column chromatography, Recrystallization	[15]
**58**	Esculin	C_15_H_16_O_9_	NMR, UHPLC-MS	Ultrasonic extraction	60% Ethanol	Silica gel column chromatography, Recrystallization	[15,22]
**59**	6,7-Dihydroxy-4-methylcoumarin	C_10_H_8_O_4_	UHPLC-Q-Orbitrap-MS	Reflux extraction method	75% Ethanol	N/A	[22]
**60**	Aesculetin-6-*O*-β-d-apiofuranosyl-(1-2)-β-Dglucopyranoside	C_20_H_24_O_13_	UHPLC-Q-Orbitrap-MS	Reflux extraction method	75% Ethanol	N/A	[22]
**61**	6,7-Di-*O*-β-d-Glucopyranosylesculetin	C_21_H_26_O_14_	NMR, MS	Reflux extraction method	Ethyl acetate	Silica gel column chromatography, Gel column chromatography, Polyamide column chromatography	[29]
**62**	7-Hydroxy-8-methoxycoumarin	C_10_H_8_O_4_	NMR, MS	Reflux extraction method	Ethyl acetate	Silica gel column chromatography, Gel column chromatography, Polyamide column chromatography	[29]
**63**	6-Hydroxy-coumarin-7-*O*-α-l-rhamnosyl-(1-6)-*O*-β-d-glucoside	C_21_H_28_O_13_	NMR, MS	Reflux extraction method	Ethyl acetate	Silica gel column chromatography, Gel column chromatography, Polyamide column chromatography	[29]
**64**	Daphneticin	C_20_H_18_O_8_	NMR, MS	Ultrasonic extraction	N/A	Liquid-liquid extraction, Silica gel column chromatography, Sephadex LH-20 column chromatography	[30]
**65**	5-Methoxy-7-hydroxymethyl coumarin	C_11_H_10_O_4_	NMR, MS	Ultrasonic extraction	N/A	Liquid-liquid extraction, Silica gel column chromatography, Sephadex LH-20 column chromatography	[30]
Terpenoids							
**66**	Yedoensins A	C_15_H_20_O_3_	HSQC, HMBC, NOESY, HR-ESI-MS	Solvent extraction method	95% Ethanol	Silica gel column chromatography, Semi-preparative HPLC	[9]
**67**	Yedoensins B	C_14_H_20_O_4_	HSQC, HMBC, NOESY, HR-ESI-MS	Solvent extraction method	95% Ethanol	Silica gel column chromatography, Semi-preparative HPLC	[9]
**68**	Versicolactone B	C_15_H_22_O_3_	HSQC, HMBC, NOESY, HR-ESI-MS	Solvent extraction method	95% Ethanol	Silica gel column chromatography, Semi-preparative HPLC	[9]
**69**	Aristolactone	C_15_H_20_O_2_	HSQC, HMBC, NOESY, HR-ESI-MS	Solvent extraction method	95% Ethanol	Silica gel column chromatography, Semi-preparative HPLC	[9]
**70**	Madolin U	C_15_H_20_O_3_	HSQC, HMBC, NOESY, HR-ESI-MS	Solvent extraction method	95% Ethanol	Silica gel column chromatography, Semi-preparative HPLC	[9]
**71**	Madolin W	C_15_H_22_O_2_	HSQC, HMBC, NOESY, HR-ESI-MS	Solvent extraction method	95% Ethanol	Silica gel column chromatography, Semi-preparative HPLC	[9]
**72**	Aristoyunnolin E	C_15_H_22_O_2_	HSQC, HMBC, NOESY, HR-ESI-MS	Solvent extraction method	95% Ethanol	Silica gel column chromatography, Semi-preparative HPLC	[9]
**73**	Isobicyclogermacrenal	C_15_H_22_O	HSQC, HMBC, NOESY, HR-ESI-MS	Solvent extraction method	95% Ethanol	Silica gel column chromatography, Semi-preparative HPLC	[9]
**74**	Madolin Y	C_15_H_22_O_3_	HSQC, HMBC, NOESY, HR-ESI-MS	Solvent extraction method	95% Ethanol	Silica gel column chromatography, Semi-preparative HPLC	[9]
**75**	Madolin R	C_15_H_24_O_2_	HSQC, HMBC, NOESY, HR-ESI-MS	Solvent extraction method	95% Ethanol	Silica gel column chromatography, Semi-preparative HPLC	[9]
**76**	Arjungenin	C_30_H_48_O_6_	UHPLC-Q-Orbitrap-MS	Reflux extraction method	75% Ethanol	N/A	[22]
**77**	Asiatic acid	C_30_H_48_O_5_	UHPLC-Q-Orbitrap-MS	Reflux extraction method	75% Ethanol	N/A	[22]
**78**	18-β-Glycyrrhetinic acid	C_30_H_46_O_4_	UHPLC-Q-Orbitrap-MS	Reflux extraction method	75% Ethanol	N/A	[22]
**79**	Ursolic acid	C_30_H_48_O_3_	UHPLC-Q-Orbitrap-MS	Reflux extraction method	75% Ethanol	N/A	[22]
**80**	Loliolide	C_11_H_16_O_3_	NMR, ESI-MS	Reflux extraction method	95% Ethanol	Silica gel column chromatography, Gel column chromatography, Polyamide column chromatography	[29]
**81**	Dehydrololiolide	C_11_H_14_O_3_	NMR, ESI-MS	Reflux extraction method	95% Ethanol	Silica gel column chromatography, Gel column chromatography, Polyamide column chromatography	[29]
**82**	Oleanolic acid	C_30_H_48_O_3_	HPLC, NMR, MS	Reflux extraction method	95% Ethanol	Silica gel column chromatography, ODS column chromatography, Sephadex LH-20 gel column chromatography, Preparative high-performance liquid chromatography	[24]
phenolic acid							
**83**	*Trans*-*p*-coumaric acid	C_9_H_8_O_3_	NMR, HPLC, MALDI-TOF MS	Sequential solvent extraction	50% Ethanol	Silica gel column chromatography, Activated carbon column chromatography, Diaion HP-20 column chromatography	[5]
**84**	*Cis*-*p*-coumaric acid	C_9_H_8_O_3_	NMR, HPLC, MALDI-TOF MS	Sequential solvent extraction	50% Ethanol	Silica gel column chromatography, Activated carbon column chromatography, Diaion HP-20 column chromatography	[5]
**85**	Vanillic acid	C_8_H_8_O_4_	NMR, HPLC, MALDI-TOF MS	Sequential solvent extraction	50% Ethanol	Silica gel column chromatography, Activated carbon column chromatography, Diaion HP-20 column chromatography	[5]
**86**	Protocatechuic acid	C_7_H_6_O_4_	NMR, EI-MS	Solvent extraction method	95% Ethanol	N/A	[8]
**87**	Neochlorogenic acid	C_16_H_18_O_9_	UHPLC-Q-Orbitrap-MS	Reflux extraction method	75% Ethanol	N/A	[22]
**88**	Chlorogenic acid	C_16_H_18_O_9_	UHPLC-Q-Orbitrap-MS	Reflux extraction method	75% Ethanol	N/A	[22]
**89**	3,5-Dicaffeoylquinic acid	C_25_H_24_O_12_	UHPLC-Q-Orbitrap-MS	Reflux extraction method	75% Ethanol	N/A	[22]
**90**	4,5-Dicaffeoylquinic acid	C_25_H_24_O_12_	UHPLC-Q-Orbitrap-MS	Reflux extraction method	75% Ethanol	N/A	[22]
**91**	3-[2-(β-d-Glucopyranosyloxy)-4-methoxyphenyl]propanoic acid	C_16_H_22_O_9_	UHPLC-Q-Orbitrap-MS	Reflux extraction method	75% Ethanol	N/A	[22]
Alkaloids							
**92**	Adenosine	C_10_H_13_N_5_O_4_	NMR, HPLC, MALDI-TOF MS	Sequential solvent extraction	50% Ethanol	Silica gel column chromatography, Activated carbon column chromatography, Diaion HP-20 column chromatography	[5]
**93**	Neoechinulin A	C_19_H_21_N_3_O_2_	ESI-MS, NMR	Solvent extraction method	95% Ethanol	Silica gel column chromatography, Sephadex LH-20 gel column chromatography	[31]
**94**	Aurantiamide acetate	C_27_H_28_N_2_O_4_	ESI-MS, NMR	Solvent extraction method	95% Ethanol	Silica gel column chromatography, Sephadex LH-20 gel column chromatography	[31]
**95**	Trichosanatine	C_27_H_28_N_2_O_4_	ESI-MS, NMR	Solvent extraction method	95% Ethanol	Silica gel column chromatography, Sephadex LH-20 gel column chromatography	[31]
**96**	Methyl indole-3-carboxylate	C_10_H_9_NO_2_	ESI-MS, NMR	Solvent extraction method	95% Ethanol	Silica gel column chromatography, Sephadex LH-20 gel column chromatography	[31]
**97**	Indole-3-carboxylic acid	C_9_H_7_NO_2_	ESI-MS, NMR	Solvent extraction method	95% Ethanol	Silica gel column chromatography, Sephadex LH-20 gel column chromatography	[31]
**98**	N-*p*-trans-Coumaroyltyramine	C_17_H_17_NO_3_	ESI-MS, NMR	Solvent extraction method	95% Ethanol	Silica gel column chromatography, Sephadex LH-20 gel column chromatography	[31]
**99**	7’-(3’,4’-Dihydroxyphenyl)-*N*-[(4-methoxyphenyl) ethyl] propenamide	C_18_H_19_NO_4_	ESI-MS, NMR	Solvent extraction method	95% Ethanol	Silica gel column chromatography, Sephadex LH-20 gel column chromatography	[31]
**100**	*N*-benzoyl-l-phenylalaninol	C_16_H_17_NO_2_	ESI-MS, NMR	Solvent extraction method	95% Ethanol	Silica gel column chromatography, Sephadex LH-20 gel column chromatography	[31]
**101**	Cannabisin F	C_36_H_36_N_2_O_8_	ESI-MS, NMR	Solvent extraction method	95% Ethanol	Silica gel column chromatography, Sephadex LH-20 gel column chromatography	[31]
**102**	*N*-benzoyl-l-*p*-hydroxy-phenylalaninol	C_16_H_17_NO_3_	MS, NMR, HPLC	Solvent extraction method	95% Ethanol	Silica gel column chromatography, Sephadex LH-20 gel column chromatography	[31]
**103**	*N*-trans-feruloyl-tyramine	C_18_H_19_NO_4_	MS, NMR	Solvent extraction method	95% Ethanol	Silica gel column chromatography, Sephadex LH-20 gel column chromatography	[31]
**104**	Aurantiamide	C_25_H_26_N_2_O_3_	MS, NMR	Solvent extraction method	95% Ethanol	Silica gel column chromatography, Sephadex LH-20 gel column chromatography	[31]
**105**	Anabellamide	C_32_H_30_N_2_O_4_	MS, NMR	Solvent extraction method	95% Ethanol	Silica gel column chromatography, Sephadex LH-20 gel column chromatography	[31]
**106**	*N*-(4-hydroxyphenethyl) hexacosanamide	C_34_H_61_NO_2_	MS, NMR	Solvent extraction method	95% Ethanol	Silica gel column chromatography, Sephadex LH-20 gel column chromatography	[31]
**107**	*N*-(4-hydroxyphenethyl) octacosanamide	C_36_H_65_NO_2_	MS, NMR	Solvent extraction method	95% Ethanol	Silica gel column chromatography, Sephadex LH-20 gel column chromatography	[31]
**108**	*N*-acetyl-1-ethyl ester glutamic acid	C_8_H_13_NO_5_	HPLC, NMR, MS	Reflux extraction method	95% Ethanol	Silica gel column chromatography, ODS column chromatography, Sephadex LH-20 gel column chromatography, Preparative high-performance liquid chromatography	[24]
**109**	*N*-acetyl glutamic acid-1-ethyl-5-methyl ester	C_10_H_17_NO_5_	HPLC, NMR, MS	Reflux extraction method	95% Ethanol	Silica gel column chromatography, ODS column chromatography, Sephadex LH-20 gel column chromatography, Preparative high-performance liquid chromatography	[24]
Others							
**110**	Lacceroic acid	C_32_H_64_O_2_	NMR, EI-MS	Solvent extraction method	95% Ethanol	N/A	[8]
**111**	Methyl palmitate	C_17_H_34_O_2_	MS, NMR	Solvent extraction method	N/A	N/A	[32]
**112**	Stearic acid	C_18_H_36_O_2_	MS, NMR	Solvent extraction method	N/A	N/A	[32]
**113**	Rel-(2α,3β)-7-*O*-methylcedrusin	C_20_H_24_O_6_	UHPLC-Q-Orbitrap-MS	Reflux extraction method	95% Ethanol	N/A	[22]
**114**	β-Sitosterol	C_29_H_50_O	NMR, ESI-MS	Solvent extraction method	75% Ethanol	Silica gel column chromatography	[23]
**115**	Daucosterol	C_35_H_60_O_6_	NMR, ESI-MS	Solvent extraction method	75% Ethanol	Silica gel column chromatography	[23]
**116**	Stigmasta-4,24(28)-dien-3-one	C_29_H_46_O	HPLC, NMR, MS	Reflux extraction method	95% Ethanol	Silica gel column chromatography, ODS column chromatography, Sephadex LH-20 gel column chromatography, Preparative high-performance liquid chromatography	[24]
**117**	Stigmasta-4,25-dien-3-one	C_29_H_46_O	HPLC, NMR, MS	Reflux extraction method	95% Ethanol	Silica gel column chromatography, ODS column chromatography, Sephadex LH-20 gel column chromatography, Preparative high-performance liquid chromatography	[24]
**118**	β-Sitostenone	C_29_H_48_O	HPLC, NMR, MS	Reflux extraction method	95% Ethanol	Silica gel column chromatography, ODS column chromatography, Sephadex LH-20 gel column chromatography, Preparative high-performance liquid chromatography	[24]
**119**	(24*R*)-3β-Hydroxy-ethylcholest-5-en-7-one	C_29_H_48_O_2_	HPLC, NMR, MS	Reflux extraction method	95% Ethanol	Silica gel column chromatography, ODS column chromatography, Sephadex LH-20 gel column chromatography, Preparative high-performance liquid chromatography	[24]
**120**	α-Tocophe rol-quinone	C_29_H_50_O_3_	HPLC, NMR, MS	Reflux extraction method	95% Ethanol	Silica gel column chromatography, ODS column chromatography, Sephadex LH-20 gel column chromatography, Preparative high-performance liquid chromatography	[24]
**121**	Triacontanol	C_30_H_62_O	MS, NMR	Solvent extraction method	N/A	N/A	[32]

N/A: information was not available.

**Table 2:** Cyclotides of *V. yedoensis* plants.

No.	Compounds	Amino Acid Sequence of Cyclotides	References
**122**	Viphi I	Cyclo-(VPCGDPSPTCVNTCNTPGCSCSWPVCTR)	[10]
**123**	Viphi J	Cyclo-(XGPVCADTCTXGTCYTAGCSCSWPVCTR)	[10]
**124**	Viphi K	Cyclo-(XGPVCGETCTXGTCYTAGCSCSWPVCTR)	[10]
**125**	Viphi L	Cyclo-(NGXPVCGETCVCYSSDPGCTCSWPVCTR)	[10]
**126**	Viphi M	Cyclo-(VPCGETCVAVGGTCNTPGCTCSWPVCTR)	[10]
**127**	Viphi N	Cyclo-(DGXPXCGETCVGGTCNTPGCSCSWPVCTR)	[10]
**128**	Viphi O	Cyclo-(DGXPVCGETCVGGTCNTPGCSCSWPVCTR)	[10]
**129**	Viphi P	Cyclo-(NGXPXCGETCVGGTCNTPGCVCSWPVCTR)	[10]
**130**	Viphi Q	Cyclo-(DGXPVCGETCTXGTCYTAGCSCSWPVCTR)	[10]
**131**	Viphi R	Cyclo-(NGXPXCGETCVGGTCDTPGCTCSWPVCTR)	[10]
**132**	Viphi S	Cyclo-(NGXPXCGETCVGDSDPTPGCTCXCPVCTR)	[10]
**133**	Viphi T	Cyclo-(DGXPVCGETCVGGTCNTPGCACSWPVCTR)	[10]
**134**	Viphi U	Cyclo-(NGXPVCEGTCVGGTCNYGGCSCSWPVCTR)	[10]
**135**	Viphi V	Cyclo-(VPCGETCVGGAVCQSNTPGCTCSWPVCTR)	[10]
**136**	Viphi W	Cyclo-(NGXPVCADTCVGGTCNTPGCACYNPVCTR)	[10]
**137**	Viphi X	Cyclo-(NGXPXCADTCVGGTCNTPGCSCSMAPVCTR)	[10]
**138**	Viphi Y	Cyclo-(VCYNGXTMCSSCVWXPCTVTAXVGCSCSDK)	[10]
**139**	Viphi Z	Cyclo-(NGXPXCEGTCVGGTCNTPGCSCSMAPVCTR)	[10]
**140**	Cycloviolacin Y1	Cyclo-(GGTIFDCGETCFLGTCYGCSCGNYGFCYGTN)	[16]
**141**	Cycloviolacin Y2	Cyclo-(GGTIFDCGESCFLGTCYTAGCSCGNWGLCYGTN)	[16]
**142**	Cycloviolacin Y3	Cyclo-(GGTIFDCGETCFLGTCYTAGCSCGNWGLCYGTN)	[16]
**143**	Cycloviolacin Y4	Cyclo-(GVPCGESCVFIPCITGVIGCSCSSNVCYLN)	[16]
**144**	Cycloviolacin Y5	Cyclo-(GIPCAESCVWIPCTVTALVGCSCSDKVCYN)	[16]
**145**	Kalata B1	Cyclo-(GLPVCGETCVGGTCNTPGCTCSWPVCTRN)	[16]
**146**	Varv A	Cyclo-GETCVGGTCNTPGCSCSWPVCTRNGLPVC	[16]
**147**	Varv E	Cyclo-(GETCVGGTCNTPGCSCSWPVCTRNGLPIC)	[16]
**148**	Viphi A	Cyclo-(CGESCVFIPCISSVIGCACKSKVCYKNGSIP)	[49]
**149**	Viphi B	Cyclo-(CGETCTIGTCYTAGCTCSWPICTRNGLPV)	[49]
**150**	Viphi C	Cyclo-(CGESCVYIPCITSVIGCSCSSKVCYINGVP)	[49]
**151**	Viphi D	Cyclo-(CGESCVFPCISSVIGCSCSSKVCYRNGIP)	[49]
**152**	Viphi E	Cyclo-(CGESCVFPCISAVIGCSCSNKVCYKNGSIP)	[49]
**153**	Viphi F	Cyclo-(CGESCVFIPCISAIIGCSCSSKVCYKNGSIP)	[49]
**154**	Viphi G	Cyclo-(CGESCVFIPCISAIIGCSCSNKVCYKNGSIP)	[49]
**155**	Viphi H	Cyclo-(CAESCVWIPCTVTAIVGCSCSWGVCYNGIP)	[49]
**156**	Viba 17	Cyclo-(CGETCVGGTCNTPGCGCSWPVCTRNGLPV)	[49]
**157**	Mram 8	Cyclo-(CGESCVFIPCLTSAIGCSCKSKVCYRNGIP)	[49]
**158**	Viba 15	Cyclo-(CGETCVGGTCNTPGCACSWPVCTRNGLPV)	[10,49]
**159**	Cycloviolacin O2	Cyclo-(CGESCVWIPCISSAIGCSCKSKVCYRNGIP)	[49]
**160**	Cycloviolacin O12	N/A	[49]
**161**	Viba 11	Cyclo-(CGESCVWIPCISGAIGCSCKSKVCYRNGIP)	[49]
**162**	Cycloviolacin VY1	Cyclo-(CGESCVFIPCITTVLGCSCSIKVCYKNGSIP)	[50]

N/A: information was not available.

**Table 3:** Summary of pharmacological activities of *V. yedoensis* extracts/compounds.

Pharmacological Activities	Component/Compound	Study Design	Models	Results/Mechanisms	Dosages	Reference
Anti-inflammatory effects	Aqueous extract of *V. yedoensis*	in vivo	Heat stress-induced broiler chickens	↓the inflammatory damage of heat stress on the spleen and thymus of broilers. ↑IgA, ↑IgG, ↑IgM, ↑ND, ↑IBD, ↓IL-1β, ↓INF-γ.	1.5%, 4.5%	[51]
	Aqueous extract of *V. yedoensis*	in vivo	LPS-induced broiler chickens	↓the activation of the NF-κB/NLRP3 signaling pathway ↑the abundance of protective bacteria ↓the number of pathogenic bacteria ↓TNF-α, ↓IL-1β, ↓IL-8, ↓NLRP3, ↓Caspase-1, ↓MyD88, ↓TLR4	0.5%, 1.5%, and 4.5%	[52]
	Anti-itching Compound of *V. yedoensis*	in vitro	RBL-2H3 mast cells	↓TNF-α, ↓IL-1β, ↓IL-6, ↓iNOS	100, 200, and 400 μg/mL	[28]
	Ethanol extract of *V. yedoensis*	in vivo	DNCB-induced atopic dermatitis-like mice	↓DNCB-stimulated AD-like lesion symptoms. ↓the ratio of CD4^+^/CD8^+^ T lymphocyte in the spleen and the number of activated macrophages stimulated by DNCB. ↓TNF-α, ↓IL-1β, ↓IL-6, ↓iNOS, ↓COX-2	5 mg/day, 10 mg/day	[22]
	Ethanol extract of *V. yedoensis* formula	in vivo	DNCB-induced atopic dermatitis-like mice	↓macrophage infiltration and promoted M2 macrophage polarization. ↓TNF-α, ↓IL-1β, ↓IL-18, ↑IL-10, ↑JAK2/STAT3 signaling pathway	150, 300 and 600 mg/kg	[14]
Anti-pyretic effects	Aqueous and ethanolic extracts of *V. yedoensis*	in vivo	LPS-induced rabbits	↓body temperature, ↓CH_50_	10, 30, 50 mg/kg	[17]
	Aqueous extract of *V. yedoensis*	in vivo	Carrageenan-induced acute inflammation ICR mice	↓body temperature ↓TNF-α, ↓IL-1β, ↓IL-6, ↓PGE_2_	5%, 35%, 50%, 80%, 100% full sunlight	[53]
Anti-virus effects	Dimethyl sulfoxide extract of *V. yedoensis*	in vitro	HIV-1, H9 cells	Does not induce interferons, does not inactivate extracellular HIV or herpes simplex virus, inhibitory to HIV	100 μg/mL	[54]
	Cycloviolacin Y5	in vitro	XTT, HIV	EC50: 0.04 µM, IC50: 1.8 µM	N/A	[16]
	Cycloviolacin VY1	in vitro	H1N1	IC_50_: 2.27 ± 0.20 μg/mL	4.00–0.25 μg/mL	[50]
Anti-cancer effects	Aqueous extract of *V. yedoensis*	in vitro	Human lung adenocarcinoma A549 cells, mouse Lewis lung cancer cells	↓the invasion of a highly metastatic human lung cancer cell line, A549 cells. ↓MMPs, ↓u-PA, ↓the DNA-binding activity of NF-κB.	10, 25, 50, 75, and 100 µg/mL	[55]
Anti-lung injury effects	Petroleum ether extract of *V. yedoensis*	in vivo	LPS-induced ALI mouse model	↓the wet-to-dry weight ratio of the lung, total cells, red blood cells, protein concentration, and myeloperoxidase activity in bronchoalveolar lavage fluid. ↓TNF-α, ↓IL-1β, ↓IL-6	2, 4 and 8 mg/kg	[56]
Anti-liver injury effects	Esculetin, prionanthoside, cichoriin, esculin, and quercetin-3-*O*-galactoside	in vivo and in vitro	in vivo: ConA-induced ILI mouse model in vitro: HepG2.2.15 cells	in vivo: ↑SOD, ↑GSH-PX, ↓ALT, ↓AST, ↓MDA, ↓TNF-α, ↓IFN-γ in vitro: ↓HBsAg, ↓HBeAg, ↓HBV DNA	in vivo: 3.00, 6.00, 12.00 g/kg/d in vitro: 0.5, 1 and 2 mM	[15]
Anti-bacterial effects	Petroleum ether and ethyl acetate extract of *V. yedoensis*	in vitro	*Bacillus subtilis* and *Pseudomonas syringae*	Petroleum ether and ethyl acetate extracts exhibited inhibitory effects against *Bacillus subtilis* and *Pseudomonas syringae*	6.25 µg/mL	[3]
	Aesculetin, 6,7-dimethoxycoumarin, scopoletin, 5-methoxy-7-hydroxymethylcoumarin	in vitro	A range of animal pathogens	↓*Staphylococcus aureus*, ↓*Escherichia coli*, ↓*Streptococcus lactis*, ↓*Streptococcus agalactiae*, ↓*Streptococcus dysgalactiae*, ↓ *Salmonella*	0.031–0.313 g/L, 0.313 -0.625 g/L	[32]
Other effects						
Anti-coagulant	Dimeresculetin, euphorbetin, esculetin	in vitro	Activated partial thromboplastin time (APTT), prothrombin time (PT), thrombin time (TT)	Esculetin and dimeresculetin had similar effects on PT and TT, while euphorbetin had more significant anticoagulant activities (except TT at a concentration of 100 µg/mL).	25, 60, and 100 µg/mL	[1]
Anti-complement	yedoensins A, yedoensins B, versicolactone B, madolin W, aristoyunnolin E, madolin Y	in vitro	Classical pathway (CP), alternative pathway (AP)	yedoensins A and versicolactone B act on C1q, C3, and C9, while madolin W, aristoyunnolin E, madolin Y interact with C1q, C3, C5, and C9	CH_50_: 0.14 to 0.37 mg/mL AP_50_: 0.32 to 0.54 mg/mL	[9]
	Alkaloid compounds of *V. yedoensis*	in vitro	Classical pathway (CP), alternative pathway (AP)	These alkaloid compounds have effects on components of the complement system, such as C1q, C2, C3, C4, C5, and C9. Different compounds in *Viola yedoensis* exert their anti-complement effects by inhibiting various targets or multiple targets of the complement system.	CH_50_: 0.12 to 0.33 g/L AP_50_: 0.22 to 0.50 g/L	[31]

(↑): improve or promote. (↓): inhibit or reduce.

## Data Availability

No new data were created or analyzed in this study. Data sharing is not applicable to this article.
